# Supplementary material for: Convalescent plasma for treatment of COVID-19: study protocol for an open randomised controlled trial in Sweden
Source: BMJ Open. 2021 Dec 7;11(12):e048337. doi: 10.1136/bmjopen-2020-048337 (PMC8655340; doi:10.1136/bmjopen-2020-048337)
Supplement: Author's manuscript [file bmjopen-2020-048337.draft_revisions.pdf]

BMJ Open is committed to open peer review. As part of this commitment we make the peer review history of every article we publish publicly available.

When an article is published we post the peer reviewers' comments and the authors' responses online. We also post the versions of the paper that were used during peer review. These are the versions that the peer review comments apply to.

The versions of the paper that follow are the versions that were submitted during the peer review process. They are not the versions of record or the final published versions. They should not be cited or distributed as the published version of this manuscript.

BMJ Open is an open access journal and the full, final, typeset and author-corrected version of record of the manuscript is available on our site with no access controls, subscription charges or pay-per-view fees (http://bmjopen.bmj.com).

If you have any questions on BMJ Open's open peer review process please email
info.bmjopen@bmj.com

BMJ Open

Convalescent plasma for treatment of COVID-19: An open randomised controlled trial in Sweden

Journal:	*BMJ Open*
Manuscript ID	bmjopen-2020-048337
Article Type:	Protocol
Date Submitted by the Author:	22-Dec-2020

Complete List of Authors:	Dillner, Joakim; Karolinska University Hospital, Center for Cervical Cancer Prevention, Karolinska University Laboratory; Karolinska Institutet, Department of Laboratory Medicine<br>Ursing, Johan; Danderyd University Hospital, Department of Infectious Diseases

Keywords:	COVID-19, Public health < INFECTIOUS DISEASES, Blood bank & transfusion medicine < PATHOLOGY, VIROLOGY, Infection control < INFECTIOUS DISEASES, INTENSIVE & CRITICAL CARE

SCHOLARONE™
Manuscripts

I, the Submitting Author has the right to grant and does grant on behalf of all authors of the Work (as defined in the below author licence), an exclusive licence and/or a non-exclusive licence for contributions from authors who are: i) UK Crown employees; ii) where BMJ has agreed a CC-BY licence shall apply, and/or iii) in accordance with the terms applicable for US Federal Government officers or employees acting as part of their official duties; on a worldwide, perpetual, irrevocable, royalty-free basis to BMJ Publishing Group Ltd ("BMJ") its licensees and where the relevant Journal is co-owned by BMJ to the co-owners of the Journal, to publish the Work in this journal and any other BMJ products and to exploit all rights, as set out in our licence.

The Submitting Author accepts and understands that any supply made under these terms is made by BMJ to the Submitting Author unless you are acting as an employee on behalf of your employer or a postgraduate student of an affiliated institution which is paying any applicable article publishing charge ("APC") for Open Access articles. Where the Submitting Author wishes to make the Work available on an Open Access basis (and intends to pay the relevant APC), the terms of reuse of such Open Access shall be governed by a Creative Commons licence – details of these licences and which Creative Commons licence will apply to this Work are set out in our licence referred to above.

Other than as permitted in any relevant BMJ Author's Self Archiving Policies, I confirm this Work has not been accepted for publication elsewhere, is not being considered for publication elsewhere and does not duplicate material already published. I confirm all authors consent to publication of this Work and authorise the granting of this licence.

For peer review only

Convalescent plasma for treatment of COVID-19: An open randomised controlled trial in Sweden

Joakim Dillner *Professor*[1], Johan Ursing *MD*[2]

1) Center for Cervical Cancer Prevention, Karolinska University Laboratory, Karolinska University Hospital and Department of Laboratory Medicine, Karolinska Institutet, Stockholm, Sweden
2) Department of Infectious Diseases, Danderyd Hospital, Stockholm, Sweden

Correspondence to: Joakim Dillner, Center for Cervical Cancer Prevention, Karolinska University Laboratory, Karolinska University Hospital and Department of Laboratory Medicine, Karolinska Institutet, S-141 86 Stockholm, Sweden

Phone: +46 72 468 24 60; E-mail: joakim.dillner@sll.se

Keywords: COVID-19, Sars-CoV-2, convalescent plasma

Word count: 2702

For peer review only - http://bmjopen.bmj.com/site/about/guidelines.xhtml

ABSTRACT

Introduction: Although there are many studies on the use of convalescent plasma (CP) for treatment of COVID-19, it is not clear i) which groups of patients may benefit, ii) what dose of plasma to give or iii) which antibody levels the plasma should contain. Previous phaseI/II studies and literature review suggest that CP should only be given to patients with viremia, that a daily infusion should be given until the patient becomes virus-free and that the neutralizing antibody titer should preferably be >1:640

Methods and analysis: An open randomised controlled trial enrolling patients with COVID-19, who must be SARS-CoV-2 positive in both airway and blood samples and admitted to a study hospital. Block randomization 2:1 is to either 200 ml convalescent plasma (preferably titre ≥1/640) daily for up to 10 days (until virus-negative in blood) plus standard care or standard care only (control arm). The primary endpoint is mortality by day 28 after study inclusion. Secondary endpoints include mortality by day 60 and doses of plasma needed to clear viremia. Assuming a reduced mortality of approximately 30% by the CP therapy and 85-88% survival in the control arm, approximately 600 participants will be enrolled to the CP therapy arm and 300 participants to the control arm.

Ethics and dissemination: Ethical approval has been granted by the Swedish Ethical Review Authority (ref 2020-06277). Results from this trial will be compiled in a clinical study report, disseminated via journal articles and communicated to stakeholders.

Trial registration number: ClinicalTrials.gov NCT04649879

For peer review only - http://bmjopen.bmj.com/site/about/guidelines.xhtml

ARTICLE SUMMARY

Strengths and limitations of this study

- A randomized trial design based on phase I and II studies that first investigated which patients to enroll (viremic patients) as well as how plasma should be characterized (high neutralizing antibody titer) and how it should be given (daily until viral clearance).
- Powered to detect a 30% mortality decrease.
- Clearance of viremia is a secondary endpoint that will be very rapid to evaluate
- Availability of high-titer convalescent plasma blood group matched to study participants may be a limiting factor
- This is an unblinded trial

For peer review only

INTRODUCTION

Corona virus disease 2019 (COVID-19) is an ongoing pandemic caused by the severe acute respiratory syndrome coronavirus 2 (SARS-CoV-2). Current treatment options are limited. Corticosteroid therapy (dexamethasone, hydrocortisone or methylprednisolone) for up to 10 days has been shown to significantly reduce mortality in severely ill COVID-19 patients, summary odds ratio, 0.66 (95% CI 0.53-0.82).[1,2] Dexamethasone also reduced the risk of progression to invasive mechanical ventilation (RR 0.76 (0.61-0.96) p=0.021). However, no improvement was seen in patients not requiring supplemental oxygen therapy and corticosteroids were associated with prolonged viral shedding.[1,3,4]

Convalescent plasma (CP) therapy is a form of immunotherapy that has been used for the treatment and prevention of infectious diseases for more than 100 years. CP was successfully used in the treatment of SARS, MERS and the 2019 H1N1 pandemic.[5-8] A meta-analysis of 32 SARS and severe influenza studies showed that CP treatment was associated with a significant reduction in the pooled odds of mortality (odds ratio, 0.25; 95% confidence interval, 0.14-0.45).[9] However, CP treatment did not significantly improve the survival of patients with Ebola disease.[10] This was possibly due to a lack of data on neutralizing antibody titres.

Early studies on CP therapy suggested that in COVID-19, CP should be given daily until the patient becomes virus-free.[11-14] In America more than 70 000 patients have received CP. A safety report on 20 000 patients indicated that CP was well tolerated and not associated with severe adverse events.[15] In a cohort of 1000 patients, mortality was significantly lower in patients receiving CP with antibody titres ≥1/640 compared to patients receiving CP with lower titres if the treatment was initiated within 72 hours of admission (data available at www.fda.gov). Though the numbers treated are large and the results suggest a beneficial

For peer review only - http://bmjopen.bmj.com/site/about/guidelines.xhtml

For peer review only

effect, the lack of controls leaves space for much uncertainty. Moreover, there are randomised studies reporting no beneficial effect, although CP therapy was not specifically given to viremic patients in these trials.[16-18] There is thus a need for a randomised clinical trial that focusses on a population at high risk of severe COVID-19 (i.e. viremic patients in early disease) and on which plasma to give and how (preferably neutralizing antibodies of at least 1:640 and one plasma bag infused daily until clearance of viremia occurs).

We found that viremia upon admission identifies patients at 7-fold increased risk of admission to intensive care and 8-fold increased risk of death.[19] CP treatment appeared to result in rapid viral clearance in a small case series and two randomized trials.[16,18,20] CP appeared to be well tolerated in a phase I study in which patients only received one dose of CP and a phase II study in which CP was given until viremia disappeared (JU, unpublished data). Moreover, mortality was 0/6, 0/18, and 6/10 in patients that were not viremic when treated, patients that were treated until viremia disappeared and patients in whom viremia continued despite treatment, respectively. The last group consisted of patients in whom treatment was stopped due to adverse events, lack of CP or because they were part of the initial phase I study in which only one dose was given. Viremic patients may thus benefit from CP and can be identified early prior to development of severe COVID-19.

Rationale

Convalescent plasma has been shown to be safe and effective for treatment of several diseases. Data indicate that it is safe for treatment of COVID-19. Preliminary data indicate that treatment should be given early prior to development of severe illness. A group at high risk of severe disease and death that has the most to benefit from CP can be easily identified by detection of viremia upon admission.[19] Phase II study data indicates that treatment should be given until SARS-CoV-2 is no longer detected in serum and the donor antibody

For peer review only - http://bmjopen.bmj.com/site/about/guidelines.xhtml

neutralization titres should be ≥1/640. A randomised controlled trial in which viremic patients are treated with CP with the equivalent of an antibody titre ≥1/640 is thus required to determine if CP can be an effective COVID-19 treatment.

Objective

The aim is to assess the efficacy, safety and tolerability of convalescent plasma for treatment of patients with COVID-19 and viremia.

For peer review only

For peer review only - http://bmjopen.bmj.com/site/about/guidelines.xhtml

METHODS AND ANALYSIS

Study design

A multicentre, open 2:1 randomised controlled clinical trial conducted in Sweden. Study sites include Karolinska University Hospital, Danderyd Hospital and the Hospital in Falun (for a complete list of study sites, visit clinicaltrials.gov NCT04649879). All Hospitals with infectious Diseases departments in Sweden have been invited to participate. The study will be conducted in accordance with the Helsinki declaration on ethical principles for medical research involving human subjects. The study is expected to take place during 2021 (2021-01-01 – 21-12-31). This study protocol is prepared using the SPIRIT guidelines.[21]

Participants

Patients with COVID-19 admitted to a study hospital will be considered for study entry. Patients will be identified by notification amongst doctors at the hospitals. Patients will be informed of the study and offered to participate if they match inclusion and exclusion criteria. An electronic (REDCap) case record form will be filled in for all screened and included patients. Upon inclusion an informed consent form will be signed. Enrolment of patients is done by dedicated physicians and expected to take place during a 12-month period. Intervention is taking place up to a maximum of 10 consecutive days and follow up will be on day 28 and day 60 after inclusion (Table 1). Adherence to study protocol will be monitored by registry linkages with the patient registry.

Eligibility criteria

Inclusion criteria for participants:

1. Age ≥18

For peer review only - http://bmjopen.bmj.com/site/about/guidelines.xhtml

For peer review only

2. Admitted to a study hospital

3. Active COVID-19 defined as symptoms + SARS CoV-2 identified from upper or lower airway samples and blood

4. Negative pregnancy test taken before inclusion and use of an acceptable effective method of contraception until treatment discontinuation if the participant is a woman of childbearing potential

5. Written informed consent after meeting with a study physician and ability and willingness to complete follow up

6. Ongoing or planned anticoagulant therapy

Exclusion criteria for participants:

1. No matching plasma donor (Exact matching in the ABO system is required)

2. Unavailability of plasma

3. Estimated glomerular filtration rate <30 (kidney failure stage III or more)

4. Pregnancy (urinary-hcg)

5. Breast feeding

6. Inability to give informed consent

The study will be conducted at wards where COVID-19 patients are treated in study hospitals. To be eligible to participate, personnel involved in the study will receive appropriate training relevant to the study.

For peer review only - http://bmjopen.bmj.com/site/about/guidelines.xhtml

Intervention

Patients will be randomised 2:1 to treatment with convalescent plasma plus standard care or standard care only. Randomisation is by random permutated blocks using REDCap or equivalent.

- Participants will receive 200 ml convalescent plasma daily until SARS-CoV-2 is no longer detectable in the blood up to a maximum of 10 CP infusions. CP will be given as a slow infusion over 2 hours. CP neutralization titre of ≥ 1/640 or an ELISA reactivity against the Spike protein of SARS-CoV-2 by the Euroimmun commercial assay >9 is desired. New antibody tests are under development and can be used instead if equivalence to neutralization or Euroimmun ELISA is demonstrated. If no high titre plasma is available, lower antibody titre plasma may be given. CP will be obtained from respective study sites blood bank. If this is not possible, CP will be available from the Department of clinical immunology and transfusion medicine Karolinska University Hospital.

- If steroid therapy has not already been initiated betamethasone 3 mg daily will be given concomitantly with plasma therapy or longer if clinically indicated but for a maximum of 10 days.

Patients will be monitored for adverse events, especially allergic reactions and impaired renal function. Patients not able to receive study treatment will receive standard care.

If an attending physician assesses that a patient is suffering from an adverse event induced by the plasma infusion and requiring treatment, the infusion will be stopped for that patient. The outcome will be classified as an adverse event necessitating treatment stop.

For peer review only

A study subject can terminate his/her participation in the study at any time without giving a reason why. Should a subject wish to do so he/she will be treated in line with standard recommendations.

Study organisers can end a patient's participation for safety reasons.

Outcomes

Primary endpoint: mortality by day 28 after inclusion into the study.

Secondary endpoints:

1. Mortality by day 60 after inclusion into the study

2. Requirement of invasive ventilation or Pao2/FiO2 $\leq$ 70 for $\geq$ 12 hours in the case of patients not eligible for intensive care

3. Adverse events

4. Dose of plasma needed to clear viremia

5. Time to clearance of viremia

Sample size

Available data indicates that CP is safe and that high titre CP can reduce mortality by approximately 30%. For that reason, a 2:1 randomisation is preferred. Randomization will be done by computer-generated random numbers. Based on available data we assume 85-88% survival in the control arm. Assuming a power of 80% and an alpha of 0,05%, 920 patients need to be enrolled.

For peer review only - http://bmjopen.bmj.com/site/about/guidelines.xhtml

Recruitment

To reach the number of patients needed to conduct the study with enough power, all hospitals in Sweden with patients acutely ill in COVID-19 are invited to participate.

Measurements

Blood samples for analysis of the following routine blood chemistry will be taken prior to start of treatment if available at respective study sites: C-reactive protein (CRP), Procalcitonin, white blood cell count (WBC), haemoglobin (Hb), Creatinine, Creatine Kinase, D-dimer, troponin, ALT, AST, LD, and bilirubin.

Blood samples for detection of SARS-CoV-2 in the blood will be taken prior to treatment start, daily during treatment and until two consecutive negative results are obtained. Serological samples will be collected for subsequent SARS-CoV-2 culture whenever PCR samples are taken.

Data collection

The following basic data will be collected and entered into electronic records (REDCap):

- Age, gender and reason for non-inclusion for screened but not included patients
- Basic patient characteristics, comorbidities, disease severity characteristics and treatment upon study entry.
- Each dose of CP and date it was given along with any observed adverse events
- Concomitant treatment with antivirals and / or corticosteroids
- The outcome of daily SARS-CoV-2 PCR until 2 consecutive negative PCRs are recorded.

For peer review only - http://bmjopen.bmj.com/site/about/guidelines.xhtml

- The need for mechanical ventilation and date when this was initiated

- For patients not eligible for intensive care: each day when PaO2/FiO2 ratio was less than 70 for ≥ 12 hours. PaO2 and FiO2 will be estimated from SO2% and O2 flow in nasal cannula, face mask or face mask with reservoir based on EPIC II data. A ratio of 70 is approximately equal to 90% SO2 with 8-9 L of Oxygen flow using a face mask with a reservoir.

- Date of death

- Survival or death by day 28 and day 60

Data is entered directly into a computerised system that includes automatic range checks.

Data analysis plan

Efficacy outcomes will be determined using cox regressions analyses, non-parametric regression analyses and survival analyses of the cohort of patients that fulfilled entry criteria but not exclusion criteria. A per protocol and intention to treat analysis will be done. Final analysis will include a description of included participants, proportions of adverse events and any serious adverse events, the proportion of participants withdrawn or lost to follow up, the cumulative success and failure rates by day 28 and day 60. Categorical variables will be compared using the $\chi 2$ test or Fisher exact test and continuous variables using quantile regression. The final data analysis will be performed when a total number of 88 outcome events have occurred. An interim analysis will be performed after 44 outcome events.

Patient and Public Involvement statement

No patient or patient organization was involved in design, recruitment or management of the study.

For peer review only - http://bmjopen.bmj.com/site/about/guidelines.xhtml

For peer review only

ETHICS AND DISSEMINATION

The trial has been registered with clinicaltrials.gov NCT04649879, and sponsor protocol number CP03. The investigational product is a blood product (not classified as a drug) and the regulatory agency is the National Inspection Authority for Health and Care (IVO), that has approved the trial. The trial has been approved by the National Ethical Review Agency (decision 2020-06277). Patients will be informed of the study verbally and in writing. Details about the trial including risks and benefits will be explained and any questions answered, including the information that all participants in the study are covered by the regular patient injury insurance. A copy of the written consent form will then be signed. If an amendment substantially alters the study design, or increases the potential risk to the patients, written informed consent must be obtained again for currently enrolled patients and must be provided to additional patients prior to their entry into the study.

The trial will be conducted in compliance with the ICH guidelines for Good Clinical Practice, the latest edition of the Declaration of Helsinki and any local regulations.

Upon inclusion an entry will be made in the patient's medical record specifying that the subject is participating in this study, if convalescent plasma treatment is given, and that written informed consent has been obtained. Any apparent side effects experienced by the subject will be assessed from the time the subject signs the informed consent and as long as the subject is part of the study, and will be reported by study site personnel either as a Baseline Event or an Adverse Event (AE). The study personnel will document any Baseline Events or AEs in the eCRF, whether observed by the investigator or reported by the subject. The safety of the different treatment arms will be assessed with regard to AEs, baseline medical conditions, and findings from the physical examination and laboratory tests. All AEs, irrespective of nature, will be followed until resolution or until end of the follow-up period.

For peer review only - http://bmjopen.bmj.com/site/about/guidelines.xhtml

The reporting period for AEs starts at inclusion and ends at the final follow-up visit 2 months after inclusion. Interim analyses will be done after inclusion of 100, 300 and 600 patients. The study will be stopped if a 30% difference in treatment outcome is detected and an Independent Data Monitoring Committee will be convened to review the data and determine if the study should be stopped or not. An external party will monitor the study regularly and ensure that the study follows GCP. Audits or inspections will be performed at the study site during or after the study. These visits may include source data verification and confidentiality documents are therefore created.

After completion of the study, a clinical study report will be prepared that will form the basis for a manuscript intended for publication in a medical journal. Data will only be reported in aggregated form, without being able to be attributed to an individual. Records of the study will be kept for 10 years after final signing of the Clinical Study Report. Data will be entered into a database by the investigators at the coordinating hospital (Danderyd Hospital). Study ID only will be used on computerised records. The key will be stored with the CRFs at Danderyd Hospital. Only the sponsor, principal investigator and participating researchers will have access to all data. Source data comprising electronic medical records will be stored at each study site.

Data sharing statement

The data constitutes sensitive data about health of human research subjects. However, pseudonymised, individual-level data that allow full replication of the results in this article will be made freely available from joakim.dillner@sll.se. The study protocol is available at clinicaltrials.gov NCT04649879.

For peer review only - http://bmjopen.bmj.com/site/about/guidelines.xhtml

Author Contributions

JD and JU conceived the study and wrote the paper. Both authors approved the final version of the manuscript.

Funding

This work was supported by the Swedish Research Council (2020-05793_3). The funding agency has had no role in the design, execution or interpretation of the study or in the decision to submit for publication.

Competing interests

None declared.

For peer review only

REFERENCES

1. Horby P, Lim WS, Emberson JR, et al. Dexamethasone in Hospitalized Patients with Covid-19 - Preliminary Report. *N Engl J Med* 2020 Jul 17:NEJMoa2021436.

2. Sterne JAC, Murthy S, Diaz JV, et al. Association Between Administration of Systemic Corticosteroids and Mortality Among Critically Ill Patients With COVID-19: A Meta-analysis. *JAMA* 2020; 324:1330-1341.

3. Arabi YM, Mandourah Y, Al-Hameed F, et al. Corticosteroid Therapy for Critically Ill Patients with Middle East Respiratory Syndrome. *Am J Respir Crit Care Med* 2018;197:757-67.

4. Stockman LJ, Bellamy R, Garner P. SARS: systematic review of treatment effects. *PLoS Med* 2006;3:e343.

5. Hung IF, To KK, Lee CK, et al. Convalescent plasma treatment reduced mortality in patients with severe pandemic influenza A (H1N1) 2009 virus infection. *Clin Infect Dis* 2011;52:447-56.

6. Zhou B, Zhong N, Guan Y. Treatment with convalescent plasma for influenza A (H5N1) infection. *N Engl J Med* 2007;357:1450-1.

7. Cheng Y, Wong R, Soo YO, et al. Use of convalescent plasma therapy in SARS patients in Hong Kong. *Eur J Clin Microbiol Infect Dis* 2005;24:44-6.

8. Ko JH, Seok H, Cho SY, et al. Challenges of convalescent plasma infusion therapy in Middle East respiratory coronavirus infection: a single centre experience. *Antivir Ther* 2018;23:617-22.

For peer review only

For peer review only - http://bmjopen.bmj.com/site/about/guidelines.xhtml

9. Mair-Jenkins J, Saavedra-Campos M, Baillie JK, et al. The effectiveness of convalescent plasma and hyperimmune immunoglobulin for the treatment of severe acute respiratory infections of viral etiology: a systematic review and exploratory meta-analysis. *J Infect Dis* 2015;211:80-90.

10. van Griensven J, Edwards T, de Lamballerie X, et al. Evaluation of Convalescent Plasma for Ebola Virus Disease in Guinea. *N Engl J Med* 2016;374:33-42.

11. Ye M, Fu D, Ren Y, et al. Treatment with convalescent plasma for COVID-19 patients in Wuhan, China. *J Med Virol* 2020;92:1890-1901.

12. Ahn JY, Sohn Y, Lee SH, et al. Use of Convalescent Plasma Therapy in Two COVID-19 Patients with Acute Respiratory Distress Syndrome in Korea. *J Korean Med Sci* 2020;35:e149.

13. Duan K, Liu B, Li C, et al. Effectiveness of convalescent plasma therapy in severe COVID-19 patients. *Proc Natl Acad Sci U S A* 2020;117:9490-9496.

14. Shen C, Wang Z, Zhao F, et al. Treatment of 5 Critically Ill Patients With COVID-19 With Convalescent Plasma. *JAMA* 2020;323:1582-1589.

15. Joyner MJ, Bruno KA, Klassen SA, et al. Safety Update: COVID-19 Convalescent Plasma in 20,000 Hospitalized Patients. *Mayo Clin Proc* 2020;95:1888-97.

16. Li L, Zhang W, Hu Y, et al. Effect of Convalescent Plasma Therapy on Time to Clinical Improvement in Patients With Severe and Life-threatening COVID-19: A Randomized Clinical Trial. *JAMA* 2020;324:460-470.

17. Simonovich VA, Burgos Pratx LD, et al. A Randomized Trial of Convalescent Plasma in Covid-19 Severe Pneumonia. *N Engl J Med* 2020 Nov 24. doi: 10.1056/NEJMoa2031304.

For peer review only

18. Agarwal A, Mukherjee A, Kumar G, et al. Convalescent plasma in the management of moderate covid-19 in adults in India: open label phase II multicentre randomised controlled trial (PLACID Trial) *BMJ* 2020;371:m3939.

19. Hagman K, Hedenstierna M, Gille-Johnson P, et al. SARS-CoV-2 RNA in serum as predictor of severe outcome in COVID-19: a retrospective cohort study. *Clin Infect Dis* 2020. doi: 10.1093/cid/ciaa1285

20. Zeng QL, Yu ZJ, Gou JJ, et al. Effect of Convalescent Plasma Therapy on Viral Shedding and Survival in Patients With Coronavirus Disease 2019. *J Infect Dis* 2020;222:38-43.

21. Chan A-W, Tetzlaff JM, Gøtzsche PC, et al. SPIRIT 2013 Explanation and Elaboration: Guidance for protocols of clinical trials. *BMJ* 2013;346:e7586.

For peer review only

For peer review only - http://bmjopen.bmj.com/site/about/guidelines.xhtml

Table 1. Planned questionnaire and sampling follow up

Time point (days)	Prior to or upon inclusion	Daily during treatment	28	60
Informed Consent	X			
CRF	X	X	X	X
Routine blood chemistry	X			
Serum sample	X	X		
Virus PCR in blood	X	X		

For peer review only

For peer review only - http://bmjopen.bmj.com/site/about/guidelines.xhtml

Reporting checklist for protocol of a clinical trial.

Based on the SPIRIT guidelines.

Instructions to authors

Complete this checklist by entering the page numbers from your manuscript where readers will find each of the items listed below.

Your article may not currently address all the items on the checklist. Please modify your text to include the missing information. If you are certain that an item does not apply, please write "n/a" and provide a short explanation.

Upload your completed checklist as an extra file when you submit to a journal.

In your methods section, say that you used the SPIRITreporting guidelines, and cite them as:

Chan A-W, Tetzlaff JM, Gøtzsche PC, Altman DG, Mann H, Berlin J, Dickersin K, Hróbjartsson A, Schulz KF, Parulekar WR, Krleža-Jerić K, Laupacis A, Moher D. SPIRIT 2013 Explanation and Elaboration: Guidance for protocols of clinical trials. BMJ. 2013;346:e7586

		Reporting Item	Page Number
**Administrative information**			
Title	#1	Descriptive title identifying the study design, population, interventions, and, if applicable, trial acronym	1
Trial registration	#2a	Trial identifier and registry name. If not yet registered, name of intended registry	2
Trial registration: data set	#2b	All items from the World Health Organization Trial Registration Data Set	1-15
Protocol version	#3	Date and version identifier	2020-12-21 version 1.2
Funding	#4	Sources and types of financial, material, and other support	15
Roles and responsibilities: contributorship	#5a	Names, affiliations, and roles of protocol contributors	1, 15

For peer review only

Roles and responsibilities: sponsor contact information	#5b	Name and contact information for the trial sponsor	1
Roles and responsibilities: sponsor and funder	#5c	Role of study sponsor and funders, if any, in study design; collection, management, analysis, and interpretation of data; writing of the report; and the decision to submit the report for publication, including whether they will have ultimate authority over any of these activities	15
Roles and responsibilities: committees	#5d	Composition, roles, and responsibilities of the coordinating centre, steering committee, endpoint adjudication committee, data management team, and other individuals or groups overseeing the trial, if applicable (see Item 21a for data monitoring committee)	14
**Introduction**			
Background and rationale	#6a	Description of research question and justification for undertaking the trial, including summary of relevant studies (published and unpublished) examining benefits and harms for each intervention	4-6
Background and rationale: choice of comparators	#6b	Explanation for choice of comparators	10
Objectives	#7	Specific objectives or hypotheses	6
Trial design	#8	Description of trial design including type of trial (eg, parallel group, crossover, factorial, single group), allocation ratio, and framework (eg, superiority, equivalence, non-inferiority, exploratory)	7
**Methods: Participants,**			

For peer review only

**interventions, and outcomes**			
Study setting	#9	Description of study settings (eg, community clinic, academic hospital) and list of countries where data will be collected. Reference to where list of study sites can be obtained	7
Eligibility criteria	#10	Inclusion and exclusion criteria for participants. If applicable, eligibility criteria for study centres and individuals who will perform the interventions (eg, surgeons, psychotherapists)	7-8
Interventions: description	#11a	Interventions for each group with sufficient detail to allow replication, including how and when they will be administered	9
Interventions: modifications	#11b	Criteria for discontinuing or modifying allocated interventions for a given trial participant (eg, drug dose change in response to harms, participant request, or improving / worsening disease)	10
Interventions: adherance	#11c	Strategies to improve adherence to intervention protocols, and any procedures for monitoring adherence (eg, drug tablet return; laboratory tests)	7
Interventions: concomitant care	#11d	Relevant concomitant care and interventions that are permitted or prohibited during the trial	8-9
Outcomes	#12	Primary, secondary, and other outcomes, including the specific measurement variable (eg, systolic blood pressure), analysis metric (eg, change from baseline, final value, time to event), method of aggregation (eg, median, proportion), and time point for each outcome. Explanation of the clinical relevance of chosen efficacy and harm outcomes is strongly recommended	10

For peer review only

Participant timeline	#13	Time schedule of enrolment, interventions (including any run-ins and washouts), assessments, and visits for participants. A schematic diagram is highly recommended (see Figure)	19
Sample size	#14	Estimated number of participants needed to achieve study objectives and how it was determined, including clinical and statistical assumptions supporting any sample size calculations	10
Recruitment	#15	Strategies for achieving adequate participant enrolment to reach target sample size	7
**Methods: Assignment of interventions (for controlled trials)**			
Allocation: sequence generation	#16a	Method of generating the allocation sequence (eg, computer-generated random numbers), and list of any factors for stratification. To reduce predictability of a random sequence, details of any planned restriction (eg, blocking) should be provided in a separate document that is unavailable to those who enrol participants or assign interventions	9
Allocation concealment mechanism	#16b	Mechanism of implementing the allocation sequence (eg, central telephone; sequentially numbered, opaque, sealed envelopes), describing any steps to conceal the sequence until interventions are assigned	9
Allocation: implementation	#16c	Who will generate the allocation sequence, who will enrol participants, and who will assign participants to interventions	7,9

For peer review only

Blinding (masking)	#17a	Who will be blinded after assignment to interventions (eg, trial participants, care providers, outcome assessors, data analysts), and how	N/A
Blinding (masking): emergency unblinding	#17b	If blinded, circumstances under which unblinding is permissible, and procedure for revealing a participant's allocated intervention during the trial	N/A
**Methods: Data collection, management, and analysis**			
Data collection plan	#18a	Plans for assessment and collection of outcome, baseline, and other trial data, including any related processes to promote data quality (eg, duplicate measurements, training of assessors) and a description of study instruments (eg, questionnaires, laboratory tests) along with their reliability and validity, if known. Reference to where data collection forms can be found, if not in the protocol	11-12
Data collection plan: retention	#18b	Plans to promote participant retention and complete follow-up, including list of any outcome data to be collected for participants who discontinue or deviate from intervention protocols	19
Data management	#19	Plans for data entry, coding, security, and storage, including any related processes to promote data quality (eg, double data entry; range checks for data values). Reference to where details of data management procedures can be found, if not in the protocol	12
Statistics: outcomes	#20a	Statistical methods for analysing primary and secondary outcomes. Reference to	12

For peer review only

		where other details of the statistical analysis plan can be found, if not in the protocol	
Statistics: additional analyses	#20b	Methods for any additional analyses (eg, subgroup and adjusted analyses)	12
Statistics: analysis population and missing data	#20c	Definition of analysis population relating to protocol non-adherence (eg, as randomised analysis), and any statistical methods to handle missing data (eg, multiple imputation)	12
**Methods: Monitoring**			
Data monitoring: formal committee	#21a	Composition of data monitoring committee (DMC); summary of its role and reporting structure; statement of whether it is independent from the sponsor and competing interests; and reference to where further details about its charter can be found, if not in the protocol. Alternatively, an explanation of why a DMC is not needed	14
Data monitoring: interim analysis	#21b	Description of any interim analyses and stopping guidelines, including who will have access to these interim results and make the final decision to terminate the trial	14
Harms	#22	Plans for collecting, assessing, reporting, and managing solicited and spontaneously reported adverse events and other unintended effects of trial interventions or trial conduct	13
Auditing	#23	Frequency and procedures for auditing trial conduct, if any, and whether the process will be independent from investigators and the sponsor	14
**Ethics and dissemination**			

For peer review only

Research ethics approval	#24	Plans for seeking research ethics committee / institutional review board (REC / IRB) approval	2<br><br>(Approval obtained 2020-12-02 from the Swedish Ethical Review Authority (ref 2020-06277).)
Protocol amendments	#25	Plans for communicating important protocol modifications (eg, changes to eligibility criteria, outcomes, analyses) to relevant parties (eg, investigators, REC / IRBs, trial participants, trial registries, journals, regulators)	13
Consent or assent	#26a	Who will obtain informed consent or assent from potential trial participants or authorised surrogates, and how (see Item 32)	7
Consent or assent: ancillary studies	#26b	Additional consent provisions for collection and use of participant data and biological specimens in ancillary studies, if applicable	N/A
Confidentiality	#27	How personal information about potential and enrolled participants will be collected, shared, and maintained in order to protect confidentiality before, during, and after the trial	14
Declaration of interests	#28	Financial and other competing interests for principal investigators for the overall trial and each study site	15
Data access	#29	Statement of who will have access to the final trial dataset, and disclosure of contractual agreements that limit such access for investigators	14
Ancillary and post trial care	#30	Provisions, if any, for ancillary and post-trial care, and for compensation to those who suffer harm from trial participation	13

For peer review only

Dissemination policy: trial results	#31a	Plans for investigators and sponsor to communicate trial results to participants, healthcare professionals, the public, and other relevant groups (eg, via publication, reporting in results databases, or other data sharing arrangements), including any publication restrictions	14
Dissemination policy: authorship	#31b	Authorship eligibility guidelines and any intended use of professional writers	N/A
Dissemination policy: reproducible research	#31c	Plans, if any, for granting public access to the full protocol, participant-level dataset, and statistical code	14
**Appendices**			
Informed consent materials	#32	Model consent form and other related documentation given to participants and authorised surrogates	Appendix 1. Study participant's information and consent form (as approved by the Swedish Ethical Review Authority (ref 2020-06277).)
Biological specimens	#33	Plans for collection, laboratory evaluation, and storage of biological specimens for genetic or molecular analysis in the current trial and for future use in ancillary studies, if applicable	Appendix 2. Information on biological specimens (as approved by the Swedish Ethical Review Authority (ref 2020-06277).)

None The SPIRIT checklist is distributed under the terms of the Creative Commons Attribution License CC-BY-ND 3.0. This checklist can be completed online using https://www.goodreports.org/, a tool made by the EQUATOR Network in collaboration with Penelope.ai

For peer review only

APPENDIX 1

Forskningspersonsinformation och informerat samtycke

Avseende studien ”Konvalescentplasma för behandling av COVID-19: en randomiserad kontrollerad studie”

Du tillfrågas härmed om att delta i en studie där plasma från personer som tillfrisknat från en SARS-CoV-2 infektion ges som tillägg till standard-behandling vid behandling av COVID-19.

Det är helt frivilligt att delta i studien och om du väljer att inte delta påverkar det inte din fortsatta behandling.

1. Bakgrund och syfte

Infektionssjukdomen COVID-19 ofta kallat corona orsakas av viruset SARS-CoV-2. Viruset orsakar allt från lindriga förkylningssymptom till en virusorsakad lunginflammation och död. Förekomst av virus i blod är en tidig markör för allvarlig sjukdom med ökad risk för död.

Konvalescentplasma är en behandling som används eller är under utvärdering i många länder. Plasma är den del av blodet som blir kvar när man tar bort de röda och vita blodkropparna. Plasma innehåller antikroppar som är en del av kroppens immunförsvar mot en infektion. Plasma från personer som haft COVID-19 (konvalescentplasma) innehåller höga nivåer av antikroppar riktade mot SARS-CoV-2. I USA har fler än 70 000 personer behandlats med sådan plasma. Behandlingen har visats vara väl tolererad och minskar möjligen risken för död. Behandlingen har därför godkänts av den Amerikanska motsvarigheten till läkemedelsverket. Om plasma verkligen gör nytta är dock inte säkerställt. Vidare är den optimala dosen oklar liksom när i sjukdomsförloppet det bör ges.

Syftet med denna studie är att utvärdera om konvalescentplasma minskar risken för död orsakad av COVID-19 när det ges till personer med virus i blod.

2. Förfrågan om deltagande

Du tillfrågas härmed om du vill delta i en denna studie där plasma från personer som har antikroppar mot SARS-CoV-2 ges till personer med akut COVID-19 sjukdom. Patienter som behandlas för coronainfektion vid Karolinska Universitetssjukhuset, Danderyds sjukhus, Södersjukhuset, Mälarsjukhuset i Eskilstuna, Gävle sjukhus, Centralsjukhuset i Karlstad eller Falu lasarett får erbjudande att delta i studien. Ansvarig för studien är Karolinska Universitetssjukhuset.

3. Hur går studien till?

Du lottas via ett datorprogram till standardbehandling eller konvalescensplasma i tillägg till standardbehandling. 2/3 av inkluderade forskningspersoner lottas till konvalescentplasma och 1/3 till standardbehandling. Cirka 20 ml blod tas vid behandlingsstart för att mäta nivån av inflammation i kroppen och identifiera din blodgrupp. Forskningspersoner som lottas till behandling får en dos konvalescensplasma à 200 ml dagligen tills virus inte längre påvisas i blod eller maximalt 10 dagar. Tio ml blod tas dagligen för att kontrollera om du har virus i blod. Uppföljning via genomgång av din journal sker dag 28 och dag 60. Den maximala totala mängden blod som tas för studien är 120 ml. Mängden blod påverkar inte din hälsa negativt. Blodprovet som tas för viruspåvisning sparas för eventuella utvidgade mikrobiella och immunologiska analyser.

For peer review only

5. Vilka är riskerna?

Plasmatransfusion är en välbeprövad klinisk rutin vid ett flertal olika sjukdomstillstånd inklusive COVID-19. Potentiella biverkningar är feber, huvudvärk, svimning eller allergisk reaktion. Andra kända biverkningar som plasma kan orsaka är övervätskning och i mycket sällsynta fall en sorts lungskada som orsakas av en specifik sorts antikropp. Konvalescentplasman som ges testas för dessa antikroppar för att säkerställa att lungskada inte uppkommer och den dagliga mängden plasma är liten, för att undvika övervätskning.

6. Finns det några fördelar?

Tidigare studier visar att konvalescensplasma möjligen minskar risken för död orsakad av COVID-19.

7. Hantering av data och sekretess

Alla data hanteras i enlighet med dataskyddsförordningen (GDPR) och sparas i 10 år. Den rättsliga grunden för detta är ditt samtycke. Du har rätt att när som helst ta tillbaka ditt samtycke.

Kliniska data kommer att pseudonymiseras genom att varje individ får en unik kod (studienummer) som länkas till personnumret. Koden som kopplar samman studienummer och personnummer kommer att förvaras i ett låst skåp i ett låst rum på Danderyds sjukhus. Kodad studiedata kommer att överföras till en studiedatabas. Dina uppgifter kommer att behandlas för statistiska beräkningar och när resultaten publiceras i medicinsk facklitteratur kommer den enskilda individen inte att kunna identifieras.

För att säkerställa att de uppgifter som samlas in och registreras är korrekta kan dessa komma att jämföras med uppgifter i din patientjournal. Detta utförs av en s.k. monitor som är en speciellt tillsatt, oberoende person med tystnadsplikt. Data kan även komma att granskas av Läkemedelsverket.

Ansvarig för dina personuppgifter är Karolinska Universitetssjukhuset. Du har rätt att kontakta oss för att få information om vilka uppgifter som behandlas om dig eller för att begära rättelse, överföring, radering eller begränsning av dina personuppgifter. Du kan även kontakta vårt dataskyddsombud på Karolinska Universitetssjukhuset (dataskyddsombud.karolinska@sll.se). Du har rätt att framföra klagomål till tillsynsmyndigheten Datainspektionen om du tycker att vi behandlar dina personuppgifter på ett felaktigt sätt.

8. Hur får jag information om studiens resultat?

Du är välkommen att höra av dig till vår huvudprövare (se kontaktuppgifter nedan) för att få information om studiens resultat.

9. Försäkring och ersättning

For peer review only

Alla deltagare i studien täcks av den ordinarie patientskadeförsäkringen. Det utgår ingen separat ersättning för att delta i studien.

10. Frivillighet

Deltagande i studien är helt frivilligt och du kan när som helst, utan särskild förklaring, avbryta. Om du avbryter kommer det inte att påverka din sedvanliga vård eller behandling.

11. Ansvariga

Forskningshuvudman:	Karolinska Universitetssjukhuset
Personuppgiftsansvarig:	Dataskyddsombudet på Karolinska Sjukhuset

Ansvarig forskare:	Professor Joakim Dillner	0724682460	Joakim.Dillner@sll.se
Klinisk Huvudprövare:	Docent Johan Ursing	08-12356760	johan.ursing@sll.se

.

For peer review only

Samtyckesformulär

Avseende studien ” Konvalescensplasma för behandling av COVID-19, en randomiserad kontrollerad studie”

- Jag bekräfta att jag har informerats både muntligen och skriftligen om denna studie, fått tillfälle att ställa frågor och fåt dem besvarade.
- Jag ger mitt samtycke till att delta i studien.
- Jag samtycker till att personuppgifter hanteras i överensstämmelse med den information jag fått enligt ovan inklusive att data kommer delas med studiemonitorn som får ta del av journaluppgifter för kontroll av data.
- Deltagande är helt frivilligt och kan när som helst, utan förklaring, avbrytas utan att detta påverkar den fortsatta sedvanliga vården.

For peer review only

Forskningspersonens namnteckning | Ort och datum

Forskningspersonens personnummer | Obs! Måste fyllas i av forskningspersonen!

Undertecknad har gått igenom och förklarat studiens syfte för ovanstående forskningsperson samt erhållit forskningspersonens samtycke. Forskningspersonen har även fått en kopia av forskningspersonsinformationen.

Prövarens namnteckning | Ort och datum

Ansökan om etikprövning – *Bilaga 2*
Beskrivning av biologiskt material

14. Beskrivning av biologiskt material

14.1. Kommer biologiskt material från människor att nyinsamlas för projektet?

Frågan avser ny insamling av biologiskt material (prover) från människor, dvs. material som samlas in specifikt för detta forskningsprojekt.

Ja

14.1.1. [Om ja 14.1] **Vilken eller vilka typer av biologiskt material ska samlas in?**

Ange vilken eller vilka typer av biologiskt material som ska samlas in för detta forskningsprojekt. Om alternativet Annat biologiskt material väljs ska dessa anges.

- ☐ Vävnad
- ☐ Blod
- ☒ Annat biologiskt material, ange vad

Serum

14.1.2. [Om ja 14.1] **Kommer det biologiska materialets ursprung gå att härleda till en enskild person, det vill säga går det att spåra eller koppla samman det biologiska materialet med den individ som materialet kommer ifrån?**

Det går att koppla samman det biologiska materialet med individen på två sätt: Antingen direkt (proverna har då märkts med identifierande uppgifter som initialer, födelsenummer eller liknande) eller genom en kodnyckel (materialet är då pseudonymiserat). Om det inte går att koppla samman det biologiska materialet med individen är materialet avidentifierat.

Ja

14.1.2.1. [Om ja 14.1.2] **Ange hur det biologiska materialet kommer att kodas.**

När ett material kodas ersätts individens identitet på materialet med en kod, och en kodnyckel tas fram. Genom kodnyckeln går det att identifiera vilken individ materialet kommer ifrån.

Proverna ges ID-nummer. ID-numret läggs in i databas som upprättas vid Karolinska Universitetslaboratoriet, där behörig personal har möjlighet att knyta det till forskningspersonens identitet.

14.1.3. [Om ja 14.1] **Hur mycket biologiskt material är planerat att samlas in?**

Ange antalet prover, antalet bitar och/eller volym som ska samlas in vid varje enskilt provtagningstillfälle. Ange även det totala antalet prover, antalet bitar och/eller den totala volymen för studien. Ange i förekommande fall även antal prover, bitar och/eller volym för varje delstudie.

Blodprov och Serumprov om 5 milliliter tas dagligen under behandlingperioden som varar 10 dagar.

14.1.4. [Om ja 14.1] **Hur länge ska det biologiska materialet vara tillgängligt för projektet?**

Ange under vilken tid forskningsprojektet kommer ha tillgång till det biologiska materialet.

2020-11-16 – 2030-11-15

14.1.5. [Om ja 14.1] **Kommer det biologiska materialet analyseras och förstöras inom sex månader efter provtagningstillfället, det vill säga är undantagsregeln tillämplig?**

Den 1 januari 2019 infördes en ny undantagsregel gällande prov som tas för forskning men som inte ska sparas i biobank. Undantagsregeln innebär att biobankslagen inte är tillämplig på prov som är avsedda för forskning och som analyseras inom sex månader efter provtagningstillfället och förstörs omedelbart efter analysen. Båda villkoren måste uppfyllas.

Även om undantagsregeln blir tillämplig i aktuellt forskningsprojekt ska alla frågor om biologiskt material besvaras med undantag för frågorna om namn och huvudman för biobanken.

Nej

For peer review only - http://bmjopen.bmj.com/site/about/guidelines.xhtml

14.1.6. [Om ja 14.1] **Ange en motivering till valet av tid som projektet kommer ha tillgång till det biologiska materialet.**

Erfarenheter av tidigare epidemier är att de ofta kommer i flera vågor, ibland under flera år. Det kan därför vara viktigt att kunna ta fram tidigare prov för parallell analys med nytagna prover. Analysmetoderna förändras snabbt och redan om något år är det troligen så stora skillnader att resultat från tidigare analyser inte kan jämföras med resultat från nya analyser på ett tillräckligt pålitligt sätt. Slutligen avser vi att publicera studien och källmaterial från publicerade studier bör vara bevarade i 10 års tid som en säkerhet för att förebygga forskningsfusk.

14.1.7. [Om ja 14.1] **Hur länge ska det biologiska materialet vara tillgängligt efter att projektet är avslutat?**

Ange under vilken tid sökanden planerar att ha tillgång till det biologiska materialet.

2020-11-16 – 2030-11-15

14.1.8. [Om ja 14.1] **Ange en motivering till valet av tid som projektet kommer ha tillgång till det biologiska materialet efter att projektet avslutats.**

Serumprov sparas för eventuell omanalys, dock längst till och med 2030-11-15

14.1.9. [Om ja 14.1] **Ange namnet på den biobank som ska ansvara för provsamlingen.**

Här avses den biobank som kommer att ansvara för den provsamling som bildas under studien, dvs. den samling som det biologiska materialet hämtas till. Ange om möjligt IVO:s registreringsnummer.

Biobank: *En samling biologiskt material från människor, förvarat för ett eller flera ändamål, samt information om detta material. En biobank kan innehålla flera provsamlingar.*

Stockholms Medicinska Biobank

14.1.10. [Om ja 14.1] **Ange huvudman för biobanken**

Ange huvudman för den biobank som ska ansvara för provsamlingen.

Huvudman: den myndighet eller organisation som juridiskt och ekonomiskt har ansvaret för viss verksamhet.

Region Stockholm

14.1.11. [Om ja 14.1] **Vilka analyser kommer att utföras på det biologiska materialet?**

Redogör för alla analyser och bearbetningar som det biologiska materialet ska genomgå.

Kvantitativ PCR och antikroppar mot Corona samt följande kemiska analyser: C-reactive protein (CRP), Procalcitonin, antal vita blodkroppar (WBC), hemoglobin (Hb), Kreatinin, Kreatine Kinas, D-dimer, Troponin, ALT, AST, LD och Bilirubin. Blodprovet efter de kemiska analyserna förstörs, medan serumprovet sparas.

14.1.12. [Om ja 14.1] **Var kommer analyserna att utföras?**

Ange var analyserna på det biologiska materialet kommer att ske. Om analyser ska ske utanför Sverige ska land/länder anges.

Olika regler gäller om prover skickas för analys inom Sverige, inom EU/ESS eller utanför EU/ESS till så kallat tredje land.

☒ I den egna verksamheten
☐ Inom Sverige
☐ Utanför Sverige, ange land/länder

Max 200 tecken

14.1.13. [Om ja 14.1] **Hur kommer det biologiska material att hanteras när analyserna är genomförda?**

Prover i en biobank som har lämnats för analys inom eller utom landet ska lämnas tillbaka till biobank i Sverige eller förstöras (kasseras) när de inte längre behövs för ändamålet. I normalfallet bör det ske inom några få år.

☒ Materialet kommer att förstöras.
☐ Materialet kommer att skickas tillbaka till biobank i Sverige.

For peer review only - http://bmjopen.bmj.com/site/about/guidelines.xhtml

14.2. Planerar projektet att använda biologiskt material från människor från en eller flera befintliga provsamlingar?

Frågan avser om man för detta forskningsprojekt kommer att begära tillgång till biologiskt material eller prover som har samlats in tidigare.

Nej

14.2.1. [Om ja 14.2] **Vilket biologiskt material är planerat att användas?**

Ange vilken typ av biologiskt material som ska användas i detta forskningsprojekt. Om alternativen "annat biologiskt material" väljs ska dessa anges.

☐ Vävnad
☐ Blod
☐ Annat biologiskt material, ange vad

[Om annat] – Max 100 tecken

14.2.2. [Om ja 14.2] **Hur mycket biologiskt material är planerat att användas?**

Ange antalet prover, bitar och/eller volymen för allt biologiskt material som är planerat att hämtas in och användas för detta projekt.

Max 2000 tecken

14.2.3. [Om ja 14.2] **Ange namnet/namnen på ansvariga biobanker och huvudmän som det biologiska materialet kommer att hämtas ifrån.**

Samtliga biobanker och huvudmän som det befintliga biologiska materialet kommer att hämtas eller begäras ut ifrån ska namnges.

Biobank: En samling biologiskt material från människor, förvarat för ett eller flera ändamål samt information om detta material. En biobank kan innehålla flera provsamlingar.

Max 2000 tecken

14.2.4. [Om ja 14.2] **Ange namnet på den biobank som kommer att ansvara för denna provsamling.**

Här avses den biobank som kommer att ansvara för den nya provsamling som bildas, dvs. den samling som det biologiska materialet hämtas till. Ange om möjligt IVO:s registreringsnummer.

Biobank: En samling biologiskt material på människor, förvarat för ett eller flera ändamål samt information om detta material. En biobank kan innehålla flera provsamlingar.

Max 200 tecken

14.2.5. [Om ja 14.2] **Ange huvudman för biobanken.**

Ange huvudman för den biobank som ska ansvara för den nya provsamlingen som bildas.

Huvudman: Den myndighet eller organisation som juridiskt och ekonomiskt har ansvaret för viss verksamhet.

Max 200 tecken

14.2.6. [Om ja 14.2] **Ange hur materialet är kodat.**

När ett material kodas ersätts individens identitet på materialet med en kod, och en kodnyckel tas fram. Genom kodnyckeln går det att identifiera vilken individ materialet kommer ifrån.

Max 2000 tecken

14.2.7. [Om ja 14.2] **Vilka analyser kommer att utföras på det biologiska materialet?**

Redogör för alla analyser och bearbetningar som det biologiska materialet ska genomgå.

Max 2000 tecken

14.2.8. [Om ja 14.2] **Var kommer analyserna att utföras?**

Ange var analyserna på det biologiska materialet kommer att ske. Om analyser ska ske utanför Sverige ska land/länder anges.

Olika regler gäller om prover skickas för analys inom Sverige, inom EU/ESS eller utanför EU/ESS till så kallat tredje land.

☐ I den egna verksamheten
☐ Inom Sverige
☐ Utanför Sverige, ange land/länder

For peer review only - http://bmjopen.bmj.com/site/about/guidelines.xhtml

[Ange land/länder] – Max 500 tecken

14.2.9. [Om ja 14.2] **Hur kommer det biologiska materialet att hanteras när analyserna är genomförda?**

Prover i en biobank som har lämnats för analys inom eller utom landet ska lämnas tillbaka till biobank i Sverige eller förstöras (kasseras) när de inte längre behövs för ändamålet. I normalfallet bör det ske inom några få år.

☐ Materialet kommer att förstöras.
☐ Materialet kommer att skickas tillbaka till biobank i Sverige.

14.2.10. [Om ja 14.2] **Hur länge ska det biologiska materialet vara tillgängligt för projektet?**

Ange under vilken tid det biologiska materialet kommer att sparas för det här forskningsprojektet.

Från och med dag månad år – Till och med dag månad år

14.2.11. [Om ja 14.2] **Ange en motivering till valet av tid som det biologiska materialet kommer vara tillgängligt för projektet.**

Max 2000 tecken

14.2.12. [Om ja 14.2] **Hur länge ska det biologiska materialet vara tillgänglig efter att projektet är avslutat?**

Ange under vilken tid sökanden planerar att ha tillgång till det biologiska materialet.

Från och med dag månad år – Till och med dag månad år

14.2.13. [Om ja 14.2] **Ange en motivering till valet av tid som det biologiska materialet ska vara tillgänglig efter att projektet är avslutat.**

Max 2000 tecken

For peer review only

For peer review only - http://bmjopen.bmj.com/site/about/guidelines.xhtml

BMJ Open

Convalescent plasma for treatment of COVID-19: An open randomised controlled trial in Sweden

Journal:	*BMJ Open*
Manuscript ID	bmjopen-2020-048337.R1
Article Type:	Protocol
Date Submitted by the Author:	11-Jun-2021
Complete List of Authors:	Dillner, Joakim; Karolinska Institutet Institutionen för laboratoriemedicin; Karolinska University Hospital, Medical Diagnostics Karolinska<br>Ursing, Johan; Danderyd University Hospital, Department of Infectious Diseases
**Primary Subject Heading**:	Infectious diseases
Secondary Subject Heading:	Infectious diseases
Keywords:	COVID-19, Public health < INFECTIOUS DISEASES, Blood bank & transfusion medicine < PATHOLOGY, VIROLOGY, Infection control < INFECTIOUS DISEASES, INTENSIVE & CRITICAL CARE

SCHOLARONE™
Manuscripts

I, the Submitting Author has the right to grant and does grant on behalf of all authors of the Work (as defined in the below author licence), an exclusive licence and/or a non-exclusive licence for contributions from authors who are: i) UK Crown employees; ii) where BMJ has agreed a CC-BY licence shall apply, and/or iii) in accordance with the terms applicable for US Federal Government officers or employees acting as part of their official duties; on a worldwide, perpetual, irrevocable, royalty-free basis to BMJ Publishing Group Ltd ("BMJ") its licensees and where the relevant Journal is co-owned by BMJ to the co-owners of the Journal, to publish the Work in this journal and any other BMJ products and to exploit all rights, as set out in our licence.

The Submitting Author accepts and understands that any supply made under these terms is made by BMJ to the Submitting Author unless you are acting as an employee on behalf of your employer or a postgraduate student of an affiliated institution which is paying any applicable article publishing charge ("APC") for Open Access articles. Where the Submitting Author wishes to make the Work available on an Open Access basis (and intends to pay the relevant APC), the terms of reuse of such Open Access shall be governed by a Creative Commons licence – details of these licences and which Creative Commons licence will apply to this Work are set out in our licence referred to above.

Other than as permitted in any relevant BMJ Author's Self Archiving Policies, I confirm this Work has not been accepted for publication elsewhere, is not being considered for publication elsewhere and does not duplicate material already published. I confirm all authors consent to publication of this Work and authorise the granting of this licence.

For peer review only

Convalescent plasma for treatment of COVID-19: An open randomised controlled trial in Sweden

Joakim Dillner *Professor*[1], Johan Ursing *MD*[2]

1) Center for Cervical Cancer Prevention, Karolinska University Laboratory, Karolinska University Hospital and Department of Laboratory Medicine, Karolinska Institutet, Stockholm, Sweden
2) Department of Infectious Diseases, Danderyd Hospital, Stockholm, Sweden

Correspondence to: Joakim Dillner, Center for Cervical Cancer Prevention, Karolinska University Laboratory, Karolinska University Hospital and Department of Laboratory Medicine, Karolinska Institutet, S-141 86 Stockholm, Sweden

Phone: +46 72 468 24 60; E-mail: Joakim.dillner@ki.se or joakim.dillner@sll.se

Keywords: COVID-19, Sars-CoV-2, convalescent plasma

Word count: 2752

For peer review only

ABSTRACT

Introduction: Although there are many studies on the use of convalescent plasma (CP) for treatment of COVID-19, it is not clear i) which groups of patients may benefit, ii) what dose of plasma to give or iii) which antibody levels the plasma should contain. Previous phaseI/II studies and literature review suggest that CP should only be given to patients with viremia, that a daily infusion should be given until the patient becomes virus-free and that the neutralizing antibody titer should preferably be >1:640

Methods and analysis: An open randomised controlled trial enrolling patients with COVID-19, who must be SARS-CoV-2 positive in both airway and blood samples and admitted to a study hospital. Block randomization 2:1 is to either 200 ml convalescent plasma (preferably titre ≥1/640) daily for up to 10 days (until virus-negative in blood) plus standard care or standard care only (control arm). The primary endpoint is mortality by day 28 after study inclusion. Secondary endpoints include mortality by day 60 and doses of plasma needed to clear viremia. Assuming a reduced mortality of approximately 30% by the CP therapy and 85-88% survival in the control arm, approximately 600 participants will be enrolled to the CP therapy arm and 300 participants to the control arm.

Ethics and dissemination: Ethical approval has been granted by the Swedish Ethical Review Authority (ref 2020-06277). Results from this trial will be compiled in a clinical study report, disseminated via journal articles and communicated to stakeholders.

Trial registration number: ClinicalTrials.gov NCT04649879

For peer review only - http://bmjopen.bmj.com/site/about/guidelines.xhtml

ARTICLE SUMMARY

Strengths and limitations of this study

- A randomized trial design based on phase I and II studies that first investigated which patients to enroll (viremic patients) as well as how plasma should be characterized (high neutralizing antibody titer) and how it should be given (daily until viral clearance).
- Powered to detect a 30% mortality decrease.
- Clearance of viremia is a secondary endpoint that will be very rapid to evaluate
- Availability of high-titer convalescent plasma blood group matched to study participants may be a limiting factor
- This is an unblinded trial

For peer review only

For peer review only - http://bmjopen.bmj.com/site/about/guidelines.xhtml

INTRODUCTION

Corona virus disease 2019 (COVID-19) is an ongoing pandemic caused by the severe acute respiratory syndrome coronavirus 2 (SARS-CoV-2). Current treatment options are limited. Corticosteroid therapy (dexamethasone, hydrocortisone or methylprednisolone) for up to 10 days has been shown to significantly reduce mortality in severely ill COVID-19 patients, summary odds ratio, 0.66 (95% CI 0.53-0.82).[1,2] Dexamethasone also reduced the risk of progression to invasive mechanical ventilation (RR 0.76 (0.61-0.96) p=0.021). However, no improvement was seen in patients not requiring supplemental oxygen therapy and corticosteroids were associated with prolonged viral shedding.[1,3,4]

Convalescent plasma (CP) therapy is a form of immunotherapy that has been used for the treatment and prevention of infectious diseases for more than 100 years. CP was successfully used in the treatment of SARS, MERS and the 2019 H1N1 pandemic.[5-8] A meta-analysis of 32 SARS and severe influenza studies showed that CP treatment was associated with a significant reduction in the pooled odds of mortality (odds ratio, 0.25; 95% confidence interval, 0.14-0.45).[9] However, CP treatment did not significantly improve the survival of patients with Ebola disease.[10] This was possibly due to a lack of data on neutralizing antibody titres.

Early studies on CP therapy suggested that in COVID-19, CP should be given daily until the patient becomes virus-free.[11-14] In America more than 70 000 patients have received CP. A safety report on 20 000 patients indicated that CP was well tolerated and not associated with severe adverse events.[15] In a cohort of 1000 patients, mortality was significantly lower in patients receiving CP with antibody titres ≥1/640 compared to patients receiving CP with lower titres if the treatment was initiated within 72 hours of admission (data available at www.fda.gov). Though the numbers treated are large and the results suggest a beneficial

For peer review only - http://bmjopen.bmj.com/site/about/guidelines.xhtml

effect, the lack of controls leaves space for much uncertainty. Moreover, there are randomised studies reporting no beneficial effect, although CP therapy was not specifically given to viremic patients in these trials.[16-18] There is thus a need for a randomised clinical trial that focusses on a population at high risk of severe COVID-19 (i.e. viremic patients in early disease) and on which plasma to give and how (preferably neutralizing antibodies of at least 1:640 and one plasma bag infused daily until clearance of viremia occurs).

We found that viremia upon admission identifies patients at 7-fold increased risk of admission to intensive care and 8-fold increased risk of death.[19] CP treatment appeared to result in rapid viral clearance in a small case series and two randomized trials.[16,18,20] CP appeared to be well tolerated in a phase I study in which patients only received one dose of CP and a phase II study in which CP was given until viremia disappeared (JU, unpublished data). Moreover, mortality was 0/6, 0/18, and 6/10 in patients that were not viremic when treated, patients that were treated until viremia disappeared and patients in whom viremia continued despite treatment, respectively. The last group consisted of patients in whom treatment was stopped due to adverse events, lack of CP or because they were part of the initial phase I study in which only one dose was given. Viremic patients may thus benefit from CP and can be identified early prior to development of severe COVID-19.

Rationale

Convalescent plasma has been shown to be safe and effective for treatment of several diseases. Data indicate that it is safe for treatment of COVID-19. Preliminary data indicate that treatment should be given early prior to development of severe illness. A group at high risk of severe disease and death that has the most to benefit from CP can be easily identified by detection of viremia upon admission.[19] Phase II study data indicates that treatment should be given until SARS-CoV-2 is no longer detected in serum and the donor antibody

For peer review only - http://bmjopen.bmj.com/site/about/guidelines.xhtml

neutralization titres should be ≥1/640. A randomised controlled trial in which viremic patients are treated with CP with the equivalent of an antibody titre ≥1/640 is thus required to determine if CP can be an effective COVID-19 treatment.

Objective

The aim is to assess the efficacy, safety and tolerability of convalescent plasma for treatment of patients with COVID-19 and viremia.

For peer review only

For peer review only - http://bmjopen.bmj.com/site/about/guidelines.xhtml

METHODS AND ANALYSIS

Study design

A multicentre, open 2:1 randomised controlled clinical trial conducted in Sweden. Study sites include Karolinska University Hospital, Danderyd Hospital and the Hospital in Falun (for a complete list of study sites, visit clinicaltrials.gov NCT04649879). All Hospitals with infectious Diseases departments in Sweden have been invited to participate. The study will be conducted in accordance with the Helsinki declaration on ethical principles for medical research involving human subjects. The study is expected to take place during 2021 (2021-01-01 – 21-12-31). This study protocol is prepared using the SPIRIT guidelines.[21]

Participants

Patients with COVID-19 admitted to a study hospital will be considered for study entry. Patients will be identified by notification amongst doctors at the hospitals. Patients will be informed of the study and offered to participate if they match inclusion and exclusion criteria. An electronic (REDCap) case record form will be filled in for all screened and included patients. Upon inclusion an informed consent form will be signed. Enrolment of patients is done by dedicated physicians and expected to take place during a 12-month period. Intervention is taking place up to a maximum of 10 consecutive days and follow up will be on day 28 and day 60 after inclusion (Table 1). Adherence to study protocol will be monitored by registry linkages with the patient registry.

Eligibility criteria

Inclusion criteria for participants:

1. Age ≥18

For peer review only - http://bmjopen.bmj.com/site/about/guidelines.xhtml

For peer review only

2. Admitted to a study hospital

3. Active COVID-19 defined as symptoms + SARS CoV-2 identified from upper or lower airway samples and blood

4. Negative pregnancy test taken before inclusion and use of an acceptable effective method of contraception until treatment discontinuation if the participant is a woman of childbearing potential

5. Written informed consent after meeting with a study physician and ability and willingness to complete follow up

6. Ongoing or planned anticoagulant therapy

Exclusion criteria for participants:

1. No matching plasma donor (Exact matching in the ABO system is required)

2. Unavailability of plasma

3. Estimated glomerular filtration rate <30 (kidney failure stage III or more)

4. Pregnancy (urinary-hcg)

5. Breast feeding

6. Inability to give informed consent

The study will be conducted at wards where COVID-19 patients are treated in study hospitals. To be eligible to participate, personnel involved in the study will receive appropriate training relevant to the study.

For peer review only - http://bmjopen.bmj.com/site/about/guidelines.xhtml

Intervention

Patients will be randomised 2:1 to treatment with convalescent plasma plus standard care or standard care only. Randomisation is by random permutated blocks using REDCap or equivalent.

- Participants will receive 200 ml convalescent plasma daily until SARS-CoV-2 is no longer detectable in the blood up to a maximum of 10 CP infusions. CP will be given as a slow infusion over 2 hours. CP neutralization titre of ≥ 1/640 (which is higher than conventionally used as high titer plasma) or an ELISA reactivity against the Spike protein of SARS-CoV-2 by the Euroimmun commercial assay >9 is desired. The in-house neutralization test is performed as described.[22]

New antibody tests are under development and can be used instead if equivalence to neutralization or Euroimmun ELISA is demonstrated. If no high titre plasma is available, lower antibody titre plasma may be given but must in no instance be lower than 4 in the Euroimmun ELISA. CP will be obtained from respective study sites blood bank. If this is not possible, CP will be available from the Department of clinical immunology and transfusion medicine Karolinska University Hospital.

- If steroid therapy has not already been initiated betamethasone 3 mg daily will be given concomitantly with plasma therapy or longer if clinically indicated but for a maximum of 10 days.

Patients will be monitored for adverse events, especially allergic reactions and impaired renal function. Patients not able to receive study treatment will receive standard care.

If an attending physician assesses that a patient is suffering from an adverse event induced by the plasma infusion and requiring treatment, the infusion will be stopped for that patient. The outcome will be classified as an adverse event necessitating treatment stop.

For peer review only - http://bmjopen.bmj.com/site/about/guidelines.xhtml

For peer review only

A study subject can terminate his/her participation in the study at any time without giving a reason why. Should a subject wish to do so he/she will be treated in line with standard recommendations.

Study organisers can end a patient's participation for safety reasons.

Outcomes

Primary endpoint: mortality by day 28 after inclusion into the study.

Secondary endpoints:

1. Mortality by day 60 after inclusion into the study

2. Requirement of invasive ventilation or Pao2/FiO2 $\leq$ 70 for $\geq$ 12 hours in the case of patients not eligible for intensive care

3. Adverse events

4. Dose of plasma needed to clear viremia

5. Time to clearance of viremia

Sample size

Available data indicates that CP is safe and that high titre CP can reduce mortality by approximately 30%. For that reason, a 2:1 randomisation is preferred. Randomization will be done by computer-generated random numbers. Based on available data we assume 85-88% survival in the control arm. Assuming a power of 80% and an alpha of 0,05%, 920 patients need to be enrolled.

For peer review only - http://bmjopen.bmj.com/site/about/guidelines.xhtml

Recruitment

To reach the number of patients needed to conduct the study with enough power, all hospitals in Sweden with patients acutely ill in COVID-19 are invited to participate.

Measurements

Blood samples for analysis of the following routine blood chemistry will be taken prior to start of treatment if available at respective study sites: C-reactive protein (CRP), Procalcitonin, white blood cell count (WBC), haemoglobin (Hb), Creatinine, Creatine Kinase, D-dimer, troponin, ALT, AST, LD, and bilirubin.

Blood samples for detection of SARS-CoV-2 in the blood will be taken prior to treatment start, daily during treatment and until two consecutive negative results are obtained. Serological samples will be collected for subsequent SARS-CoV-2 culture whenever PCR samples are taken.

Data collection

The following basic data will be collected and entered into electronic records (REDCap):

- Age, gender and reason for non-inclusion for screened but not included patients
- Date of symtom onset and date of start of CP theraphy will be recorded and used in subgroup analyses.
- Basic patient characteristics, comorbidities, disease severity characteristics and treatment upon study entry.
- Each dose of CP and date it was given along with any observed adverse events
- Concomitant treatment with antivirals and / or corticosteroids

For peer review only - http://bmjopen.bmj.com/site/about/guidelines.xhtml

- The outcome of daily SARS-CoV-2 PCR until 2 consecutive negative PCRs are recorded.
- The need for mechanical ventilation and date when this was initiated
- For patients not eligible for intensive care: each day when PaO2/FiO2 ratio was less than 70 for ≥ 12 hours. PaO2 and FiO2 will be estimated from SO2% and O2 flow in nasal cannula, face mask or face mask with reservoir based on EPIC II data. A ratio of 70 is approximately equal to 90% SO2 with 8-9 L of Oxygen flow using a face mask with a reservoir.
- Date of death
- Survival or death by day 28 and day 60

Data is entered directly into a computerised system that includes automatic range checks.

Data analysis plan

Efficacy outcomes will be determined using cox regressions analyses, non-parametric regression analyses and survival analyses of the cohort of patients that fulfilled entry criteria but not exclusion criteria. A per protocol and intention to treat analysis will be done. Final analysis will include a description of included participants, proportions of adverse events and any serious adverse events, the proportion of participants withdrawn or lost to follow up, the cumulative success and failure rates by day 28 and day 60. Categorical variables will be compared using the $\chi 2$ test or Fisher exact test and continuous variables using quantile regression. The final data analysis will be performed when a total number of 88 outcome events have occurred. An interim analysis will be performed after 44 outcome events.

Patient and Public Involvement statement

For peer review only - http://bmjopen.bmj.com/site/about/guidelines.xhtml

No patient or patient organization was involved in design, recruitment or management of the study.

ETHICS AND DISSEMINATION

The trial has been registered with clinicaltrials.gov NCT04649879, and sponsor protocol number CP03. The investigational product is a blood product (not classified as a drug) and the regulatory agency is the National Inspection Authority for Health and Care (IVO), that has approved the trial. The trial has been approved by the National Ethical Review Agency (decision 2020-06277). Patients will be informed of the study verbally and in writing. Details about the trial including risks and benefits will be explained and any questions answered, including the information that all participants in the study are covered by the regular patient injury insurance. A copy of the written consent form will then be signed. If an amendment substantially alters the study design, or increases the potential risk to the patients, written informed consent must be obtained again for currently enrolled patients and must be provided to additional patients prior to their entry into the study.

The trial will be conducted in compliance with the ICH guidelines for Good Clinical Practice, the latest edition of the Declaration of Helsinki and any local regulations.

Upon inclusion an entry will be made in the patient's medical record specifying that the subject is participating in this study, if convalescent plasma treatment is given, and that written informed consent has been obtained. Any apparent side effects experienced by the subject will be assessed from the time the subject signs the informed consent and as long as the subject is part of the study, and will be reported by study site personnel either as a Baseline Event or an Adverse Event (AE). The study personnel will document any Baseline Events or AEs in the eCRF, whether observed by the investigator or reported by the subject. The safety of the different treatment arms will be assessed with regard to AEs, baseline

For peer review only

For peer review only - http://bmjopen.bmj.com/site/about/guidelines.xhtml

medical conditions, and findings from the physical examination and laboratory tests. All AEs, irrespective of nature, will be followed until resolution or until end of the follow-up period.

The reporting period for AEs starts at inclusion and ends at the final follow-up visit 2 months after inclusion. Interim analyses will be done after inclusion of 100, 300 and 600 patients. The study will be stopped if a 30% difference in treatment outcome is detected and an Independent Data Monitoring Committee will be convened to review the data and determine if the study should be stopped or not. An external party will monitor the study regularly and ensure that the study follows GCP. Audits or inspections will be performed at the study site during or after the study. These visits may include source data verification and confidentiality documents are therefore created.

After completion of the study, a clinical study report will be prepared that will form the basis for a manuscript intended for publication in a medical journal. Data will only be reported in aggregated form, without being able to be attributed to an individual. Records of the study will be kept for 10 years after final signing of the Clinical Study Report. Data will be entered into a database by the investigators at the coordinating hospital (Danderyd Hospital). Study ID only will be used on computerised records. The key will be stored with the CRFs at Danderyd Hospital. Only the sponsor, principal investigator and participating researchers will have access to all data. Source data comprising electronic medical records will be stored at each study site.

Data sharing statement

The data constitutes sensitive data about health of human research subjects. However, pseudonymised, individual-level data that allow full replication of the results in this article will be made freely available from joakim.dillner@sll.se. The study protocol is available at clinicaltrials.gov NCT04649879.

For peer review only - http://bmjopen.bmj.com/site/about/guidelines.xhtml

Author Contributions

JD and JU conceived the study and wrote the paper. Both authors approved the final version of the manuscript.

Funding

This work was supported by the Swedish Research Council (2020-05793_3). The funding agency has had no role in the design, execution or interpretation of the study or in the decision to submit for publication.

Competing interests

None declared.

For peer review only

REFERENCES

1. Horby P, Lim WS, Emberson JR, et al. Dexamethasone in Hospitalized Patients with Covid-19 - Preliminary Report. *N Engl J Med* 2020 Jul 17:NEJMoa2021436.

2. Sterne JAC, Murthy S, Diaz JV, et al. Association Between Administration of Systemic Corticosteroids and Mortality Among Critically Ill Patients With COVID-19: A Meta-analysis. *JAMA* 2020; 324:1330-1341.

3. Arabi YM, Mandourah Y, Al-Hameed F, et al. Corticosteroid Therapy for Critically Ill Patients with Middle East Respiratory Syndrome. *Am J Respir Crit Care Med* 2018;197:757-67.

4. Stockman LJ, Bellamy R, Garner P. SARS: systematic review of treatment effects. *PLoS Med* 2006;3:e343.

5. Hung IF, To KK, Lee CK, et al. Convalescent plasma treatment reduced mortality in patients with severe pandemic influenza A (H1N1) 2009 virus infection. *Clin Infect Dis* 2011;52:447-56.

6. Zhou B, Zhong N, Guan Y. Treatment with convalescent plasma for influenza A (H5N1) infection. *N Engl J Med* 2007;357:1450-1.

7. Cheng Y, Wong R, Soo YO, et al. Use of convalescent plasma therapy in SARS patients in Hong Kong. *Eur J Clin Microbiol Infect Dis* 2005;24:44-6.

8. Ko JH, Seok H, Cho SY, et al. Challenges of convalescent plasma infusion therapy in Middle East respiratory coronavirus infection: a single centre experience. *Antivir Ther* 2018;23:617-22.

For peer review only - http://bmjopen.bmj.com/site/about/guidelines.xhtml

9. Mair-Jenkins J, Saavedra-Campos M, Baillie JK, et al. The effectiveness of convalescent plasma and hyperimmune immunoglobulin for the treatment of severe acute respiratory infections of viral etiology: a systematic review and exploratory meta-analysis. *J Infect Dis* 2015;211:80-90.

10. van Griensven J, Edwards T, de Lamballerie X, et al. Evaluation of Convalescent Plasma for Ebola Virus Disease in Guinea. *N Engl J Med* 2016;374:33-42.

11. Ye M, Fu D, Ren Y, et al. Treatment with convalescent plasma for COVID-19 patients in Wuhan, China. *J Med Virol* 2020;92:1890-1901.

12. Ahn JY, Sohn Y, Lee SH, et al. Use of Convalescent Plasma Therapy in Two COVID-19 Patients with Acute Respiratory Distress Syndrome in Korea. *J Korean Med Sci* 2020;35:e149.

13. Duan K, Liu B, Li C, et al. Effectiveness of convalescent plasma therapy in severe COVID-19 patients. *Proc Natl Acad Sci U S A* 2020;117:9490-9496.

14. Shen C, Wang Z, Zhao F, et al. Treatment of 5 Critically Ill Patients With COVID-19 With Convalescent Plasma. *JAMA* 2020;323:1582-1589.

15. Joyner MJ, Bruno KA, Klassen SA, et al. Safety Update: COVID-19 Convalescent Plasma in 20,000 Hospitalized Patients. *Mayo Clin Proc* 2020;95:1888-97.

16. Li L, Zhang W, Hu Y, et al. Effect of Convalescent Plasma Therapy on Time to Clinical Improvement in Patients With Severe and Life-threatening COVID-19: A Randomized Clinical Trial. *JAMA* 2020;324:460-470.

17. Simonovich VA, Burgos Pratx LD, et al. A Randomized Trial of Convalescent Plasma in Covid-19 Severe Pneumonia. *N Engl J Med* 2020 Nov 24. doi: 10.1056/NEJMoa2031304.

For peer review only - http://bmjopen.bmj.com/site/about/guidelines.xhtml

For peer review only

18. Agarwal A, Mukherjee A, Kumar G, et al. Convalescent plasma in the management of moderate covid-19 in adults in India: open label phase II multicentre randomised controlled trial (PLACID Trial) *BMJ* 2020;371:m3939.

19. Hagman K, Hedenstierna M, Gille-Johnson P, et al. SARS-CoV-2 RNA in serum as predictor of severe outcome in COVID-19: a retrospective cohort study. *Clin Infect Dis* 2020. doi: 10.1093/cid/ciaa1285

20. Zeng QL, Yu ZJ, Gou JJ, et al. Effect of Convalescent Plasma Therapy on Viral Shedding and Survival in Patients With Coronavirus Disease 2019. *J Infect Dis* 2020;222:38-43.

21. Chan A-W, Tetzlaff JM, Gøtzsche PC, et al. SPIRIT 2013 Explanation and Elaboration: Guidance for protocols of clinical trials. *BMJ* 2013;346:e7586.

22. Varnaitė R, García M, Glans H, et al. Expansion of SARS-CoV-2-Specific Antibody-Secreting Cells and Generation of Neutralizing Antibodies in Hospitalized COVID-19 Patients. *J Immunol.* 2020;205:2437-2446.

For peer review only

For peer review only - http://bmjopen.bmj.com/site/about/guidelines.xhtml

Table 1. Planned questionnaire and sampling follow up

Time point (days)	Prior to or upon inclusion	Daily during treatment	28	60
**Informed Consent**	X			
**CRF**	X	**X**	**X**	**X**
**Routine blood chemistry**	X			
**Serum sample**	X	X		
**Virus PCR in blood**	X	X		

For peer review only

Reporting checklist for protocol of a clinical trial.

Based on the SPIRIT guidelines.

Instructions to authors

Complete this checklist by entering the page numbers from your manuscript where readers will find each of the items listed below.

Your article may not currently address all the items on the checklist. Please modify your text to include the missing information. If you are certain that an item does not apply, please write "n/a" and provide a short explanation.

Upload your completed checklist as an extra file when you submit to a journal.

In your methods section, say that you used the SPIRITreporting guidelines, and cite them as:

Chan A-W, Tetzlaff JM, Gøtzsche PC, Altman DG, Mann H, Berlin J, Dickersin K, Hróbjartsson A, Schulz KF, Parulekar WR, Krleža-Jerić K, Laupacis A, Moher D. SPIRIT 2013 Explanation and Elaboration: Guidance for protocols of clinical trials. BMJ. 2013;346:e7586

		Reporting Item	Page Number
**Administrative information**			
Title	#1	Descriptive title identifying the study design, population, interventions, and, if applicable, trial acronym	1
Trial registration	#2a	Trial identifier and registry name. If not yet registered, name of intended registry	2
Trial registration: data set	#2b	All items from the World Health Organization Trial Registration Data Set	1-15
Protocol version	#3	Date and version identifier	2020-12-21 version 1.2
Funding	#4	Sources and types of financial, material, and other support	15
Roles and responsibilities: contributorship	#5a	Names, affiliations, and roles of protocol contributors	1, 15

For peer review only

Roles and responsibilities: sponsor contact information	#5b	Name and contact information for the trial sponsor	1
Roles and responsibilities: sponsor and funder	#5c	Role of study sponsor and funders, if any, in study design; collection, management, analysis, and interpretation of data; writing of the report; and the decision to submit the report for publication, including whether they will have ultimate authority over any of these activities	15
Roles and responsibilities: committees	#5d	Composition, roles, and responsibilities of the coordinating centre, steering committee, endpoint adjudication committee, data management team, and other individuals or groups overseeing the trial, if applicable (see Item 21a for data monitoring committee)	14
**Introduction**			
Background and rationale	#6a	Description of research question and justification for undertaking the trial, including summary of relevant studies (published and unpublished) examining benefits and harms for each intervention	4-6
Background and rationale: choice of comparators	#6b	Explanation for choice of comparators	10
Objectives	#7	Specific objectives or hypotheses	6
Trial design	#8	Description of trial design including type of trial (eg, parallel group, crossover, factorial, single group), allocation ratio, and framework (eg, superiority, equivalence, non-inferiority, exploratory)	7
**Methods: Participants,**			

For peer review only

**interventions, and outcomes**			
Study setting	#9	Description of study settings (eg, community clinic, academic hospital) and list of countries where data will be collected. Reference to where list of study sites can be obtained	7
Eligibility criteria	#10	Inclusion and exclusion criteria for participants. If applicable, eligibility criteria for study centres and individuals who will perform the interventions (eg, surgeons, psychotherapists)	7-8
Interventions: description	#11a	Interventions for each group with sufficient detail to allow replication, including how and when they will be administered	9
Interventions: modifications	#11b	Criteria for discontinuing or modifying allocated interventions for a given trial participant (eg, drug dose change in response to harms, participant request, or improving / worsening disease)	10
Interventions: adherance	#11c	Strategies to improve adherence to intervention protocols, and any procedures for monitoring adherence (eg, drug tablet return; laboratory tests)	7
Interventions: concomitant care	#11d	Relevant concomitant care and interventions that are permitted or prohibited during the trial	8-9
Outcomes	#12	Primary, secondary, and other outcomes, including the specific measurement variable (eg, systolic blood pressure), analysis metric (eg, change from baseline, final value, time to event), method of aggregation (eg, median, proportion), and time point for each outcome. Explanation of the clinical relevance of chosen efficacy and harm outcomes is strongly recommended	10

For peer review only

Participant timeline	#13	Time schedule of enrolment, interventions (including any run-ins and washouts), assessments, and visits for participants. A schematic diagram is highly recommended (see Figure)	19
Sample size	#14	Estimated number of participants needed to achieve study objectives and how it was determined, including clinical and statistical assumptions supporting any sample size calculations	10
Recruitment	#15	Strategies for achieving adequate participant enrolment to reach target sample size	7
**Methods: Assignment of interventions (for controlled trials)**			
Allocation: sequence generation	#16a	Method of generating the allocation sequence (eg, computer-generated random numbers), and list of any factors for stratification. To reduce predictability of a random sequence, details of any planned restriction (eg, blocking) should be provided in a separate document that is unavailable to those who enrol participants or assign interventions	9
Allocation concealment mechanism	#16b	Mechanism of implementing the allocation sequence (eg, central telephone; sequentially numbered, opaque, sealed envelopes), describing any steps to conceal the sequence until interventions are assigned	9
Allocation: implementation	#16c	Who will generate the allocation sequence, who will enrol participants, and who will assign participants to interventions	7,9

For peer review only

Blinding (masking)	#17a	Who will be blinded after assignment to interventions (eg, trial participants, care providers, outcome assessors, data analysts), and how	N/A
Blinding (masking): emergency unblinding	#17b	If blinded, circumstances under which unblinding is permissible, and procedure for revealing a participant's allocated intervention during the trial	N/A
**Methods: Data collection, management, and analysis**			
Data collection plan	#18a	Plans for assessment and collection of outcome, baseline, and other trial data, including any related processes to promote data quality (eg, duplicate measurements, training of assessors) and a description of study instruments (eg, questionnaires, laboratory tests) along with their reliability and validity, if known. Reference to where data collection forms can be found, if not in the protocol	11-12
Data collection plan: retention	#18b	Plans to promote participant retention and complete follow-up, including list of any outcome data to be collected for participants who discontinue or deviate from intervention protocols	19
Data management	#19	Plans for data entry, coding, security, and storage, including any related processes to promote data quality (eg, double data entry; range checks for data values). Reference to where details of data management procedures can be found, if not in the protocol	12
Statistics: outcomes	#20a	Statistical methods for analysing primary and secondary outcomes. Reference to	12

For peer review only

		where other details of the statistical analysis plan can be found, if not in the protocol	
Statistics: additional analyses	#20b	Methods for any additional analyses (eg, subgroup and adjusted analyses)	12
Statistics: analysis population and missing data	#20c	Definition of analysis population relating to protocol non-adherence (eg, as randomised analysis), and any statistical methods to handle missing data (eg, multiple imputation)	12
**Methods: Monitoring**			
Data monitoring: formal committee	#21a	Composition of data monitoring committee (DMC); summary of its role and reporting structure; statement of whether it is independent from the sponsor and competing interests; and reference to where further details about its charter can be found, if not in the protocol. Alternatively, an explanation of why a DMC is not needed	14
Data monitoring: interim analysis	#21b	Description of any interim analyses and stopping guidelines, including who will have access to these interim results and make the final decision to terminate the trial	14
Harms	#22	Plans for collecting, assessing, reporting, and managing solicited and spontaneously reported adverse events and other unintended effects of trial interventions or trial conduct	13
Auditing	#23	Frequency and procedures for auditing trial conduct, if any, and whether the process will be independent from investigators and the sponsor	14
**Ethics and dissemination**			

For peer review only

Research ethics approval	#24	Plans for seeking research ethics committee / institutional review board (REC / IRB) approval	2<br><br>(Approval obtained 2020-12-02 from the Swedish Ethical Review Authority (ref 2020-06277).)
Protocol amendments	#25	Plans for communicating important protocol modifications (eg, changes to eligibility criteria, outcomes, analyses) to relevant parties (eg, investigators, REC / IRBs, trial participants, trial registries, journals, regulators)	13
Consent or assent	#26a	Who will obtain informed consent or assent from potential trial participants or authorised surrogates, and how (see Item 32)	7
Consent or assent: ancillary studies	#26b	Additional consent provisions for collection and use of participant data and biological specimens in ancillary studies, if applicable	N/A
Confidentiality	#27	How personal information about potential and enrolled participants will be collected, shared, and maintained in order to protect confidentiality before, during, and after the trial	14
Declaration of interests	#28	Financial and other competing interests for principal investigators for the overall trial and each study site	15
Data access	#29	Statement of who will have access to the final trial dataset, and disclosure of contractual agreements that limit such access for investigators	14
Ancillary and post trial care	#30	Provisions, if any, for ancillary and post-trial care, and for compensation to those who suffer harm from trial participation	13

For peer review only

Dissemination policy: trial results	#31a	Plans for investigators and sponsor to communicate trial results to participants, healthcare professionals, the public, and other relevant groups (eg, via publication, reporting in results databases, or other data sharing arrangements), including any publication restrictions	14
Dissemination policy: authorship	#31b	Authorship eligibility guidelines and any intended use of professional writers	N/A
Dissemination policy: reproducible research	#31c	Plans, if any, for granting public access to the full protocol, participant-level dataset, and statistical code	14
**Appendices**			
Informed consent materials	#32	Model consent form and other related documentation given to participants and authorised surrogates	Appendix 1. Study participant's information and consent form (as approved by the Swedish Ethical Review Authority (ref 2020-06277).)
Biological specimens	#33	Plans for collection, laboratory evaluation, and storage of biological specimens for genetic or molecular analysis in the current trial and for future use in ancillary studies, if applicable	Appendix 2. Information on biological specimens (as approved by the Swedish Ethical Review Authority (ref 2020-06277).)

None The SPIRIT checklist is distributed under the terms of the Creative Commons Attribution License CC-BY-ND 3.0. This checklist can be completed online using https://www.goodreports.org/, a tool made by the EQUATOR Network in collaboration with Penelope.ai

APPENDIX 1

Forskningspersonsinformation och informerat samtycke

Avseende studien ”Konvalescentplasma för behandling av COVID-19: en randomiserad kontrollerad studie”

Du tillfrågas härmed om att delta i en studie där plasma från personer som tillfrisknat från en SARS-CoV-2 infektion ges som tillägg till standard-behandling vid behandling av COVID-19.

Det är helt frivilligt att delta i studien och om du väljer att inte delta påverkar det inte din fortsatta behandling.

1. Bakgrund och syfte

Infektionssjukdomen COVID-19 ofta kallat corona orsakas av viruset SARS-CoV-2. Viruset orsakar allt från lindriga förkylningssymptom till en virusorsakad lunginflammation och död. Förekomst av virus i blod är en tidig markör för allvarlig sjukdom med ökad risk för död.

Konvalescentplasma är en behandling som används eller är under utvärdering i många länder. Plasma är den del av blodet som blir kvar när man tar bort de röda och vita blodkropparna. Plasma innehåller antikroppar som är en del av kroppens immunförsvar mot en infektion. Plasma från personer som haft COVID-19 (konvalescentplasma) innehåller höga nivåer av antikroppar riktade mot SARS-CoV-2. I USA har fler än 70 000 personer behandlats med sådan plasma. Behandlingen har visats vara väl tolererad och minskar möjligen risken för död. Behandlingen har därför godkänts av den Amerikanska motsvarigheten till läkemedelsverket. Om plasma verkligen gör nytta är dock inte säkerställt. Vidare är den optimala dosen oklar liksom när i sjukdomsförloppet det bör ges.

Syftet med denna studie är att utvärdera om konvalescentplasma minskar risken för död orsakad av COVID-19 när det ges till personer med virus i blod.

2. Förfrågan om deltagande

Du tillfrågas härmed om du vill delta i en denna studie där plasma från personer som har antikroppar mot SARS-CoV-2 ges till personer med akut COVID-19 sjukdom. Patienter som behandlas för coronainfektion vid Karolinska Universitetssjukhuset, Danderyds sjukhus, Södersjukhuset, Mälarsjukhuset i Eskilstuna, Gävle sjukhus, Centralsjukhuset i Karlstad eller Falu lasarett får erbjudande att delta i studien. Ansvarig för studien är Karolinska Universitetssjukhuset.

3. Hur går studien till?

Du lottas via ett datorprogram till standardbehandling eller konvalescensplasma i tillägg till standardbehandling. 2/3 av inkluderade forskningspersoner lottas till konvalescentplasma och 1/3 till standardbehandling. Cirka 20 ml blod tas vid behandlingsstart för att mäta nivån av inflammation i kroppen och identifiera din blodgrupp. Forskningspersoner som lottas till behandling får en dos konvalescensplasma à 200 ml dagligen tills virus inte längre påvisas i blod eller maximalt 10 dagar. Tio ml blod tas dagligen för att kontrollera om du har virus i blod. Uppföljning via genomgång av din journal sker dag 28 och dag 60. Den maximala totala mängden blod som tas för studien är 120 ml. Mängden blod påverkar inte din hälsa negativt. Blodprovet som tas för viruspåvisning sparas för eventuella utvidgade mikrobiella och immunologiska analyser.

For peer review only

5. Vilka är riskerna?

Plasmatransfusion är en välbeprövad klinisk rutin vid ett flertal olika sjukdomstillstånd inklusive COVID-19. Potentiella biverkningar är feber, huvudvärk, svimning eller allergisk reaktion. Andra kända biverkningar som plasma kan orsaka är övervätskning och i mycket sällsynta fall en sorts lungskada som orsakas av en specifik sorts antikropp. Konvalescentplasman som ges testas för dessa antikroppar för att säkerställa att lungskada inte uppkommer och den dagliga mängden plasma är liten, för att undvika övervätskning.

6. Finns det några fördelar?

Tidigare studier visar att konvalescensplasma möjligen minskar risken för död orsakad av COVID-19.

7. Hantering av data och sekretess

Alla data hanteras i enlighet med dataskyddsförordningen (GDPR) och sparas i 10 år. Den rättsliga grunden för detta är ditt samtycke. Du har rätt att när som helst ta tillbaka ditt samtycke.

Kliniska data kommer att pseudonymiseras genom att varje individ får en unik kod (studienummer) som länkas till personnumret. Koden som kopplar samman studienummer och personnummer kommer att förvaras i ett låst skåp i ett låst rum på Danderyds sjukhus. Kodad studiedata kommer att överföras till en studiedatabas. Dina uppgifter kommer att behandlas för statistiska beräkningar och när resultaten publiceras i medicinsk facklitteratur kommer den enskilda individen inte att kunna identifieras.

För att säkerställa att de uppgifter som samlas in och registreras är korrekta kan dessa komma att jämföras med uppgifter i din patientjournal. Detta utförs av en s.k. monitor som är en speciellt tillsatt, oberoende person med tystnadsplikt. Data kan även komma att granskas av Läkemedelsverket.

Ansvarig för dina personuppgifter är Karolinska Universitetssjukhuset. Du har rätt att kontakta oss för att få information om vilka uppgifter som behandlas om dig eller för att begära rättelse, överföring, radering eller begränsning av dina personuppgifter. Du kan även kontakta vårt dataskyddsombud på Karolinska Universitetssjukhuset (dataskyddsombud.karolinska@sll.se). Du har rätt att framföra klagomål till tillsynsmyndigheten Datainspektionen om du tycker att vi behandlar dina personuppgifter på ett felaktigt sätt.

8. Hur får jag information om studiens resultat?

Du är välkommen att höra av dig till vår huvudprövare (se kontaktuppgifter nedan) för att få information om studiens resultat.

9. Försäkring och ersättning

For peer review only

Alla deltagare i studien täcks av den ordinarie patientskadeförsäkringen. Det utgår ingen separat ersättning för att delta i studien.

10. Frivillighet

Deltagande i studien är helt frivilligt och du kan när som helst, utan särskild förklaring, avbryta. Om du avbryter kommer det inte att påverka din sedvanliga vård eller behandling.

11. Ansvariga

Forskningshuvudman:	Karolinska Universitetssjukhuset		
Personuppgiftsansvarig:	Dataskyddsombudet på Karolinska Sjukhuset		
Ansvarig forskare:	Professor Joakim Dillner	0724682460	Joakim.Dillner@sll.se
Klinisk Huvudprövare:	Docent Johan Ursing	08-12356760	johan.ursing@sll.se

.

For peer review only

Samtyckesformulär

Avseende studien " Konvalescensplasma för behandling av COVID-19, en randomiserad kontrollerad studie"

- Jag bekräfta att jag har informerats både muntligen och skriftligen om denna studie, fått tillfälle att ställa frågor och fåt dem besvarade.
- Jag ger mitt samtycke till att delta i studien.
- Jag samtycker till att personuppgifter hanteras i överensstämmelse med den information jag fått enligt ovan inklusive att data kommer delas med studiemonitorn som får ta del av journaluppgifter för kontroll av data.
- Deltagande är helt frivilligt och kan när som helst, utan förklaring, avbrytas utan att detta påverkar den fortsatta sedvanliga vården.

Forskningspersonens namnteckning

Ort och datum

Forskningspersonens personnummer

Obs! Måste fyllas i av forskningspersonen!

Undertecknad har gått igenom och förklarat studiens syfte för ovanstående forskningsperson samt erhållit forskningspersonens samtycke. Forskningspersonen har även fått en kopia av forskningspersonsinformationen.

Prövarens namnteckning

Ort och datum

For peer review only

APPENDIX 2

Ansökan om etikprövning – *Bilaga 2*
Beskrivning av biologiskt material

14. Beskrivning av biologiskt material

14.1. Kommer biologiskt material från människor att nyinsamlas för projektet?

Frågan avser ny insamling av biologiskt material (prover) från människor, dvs. material som samlas in specifikt för detta forskningsprojekt.

Ja

14.1.1. [Om ja 14.1] **Vilken eller vilka typer av biologiskt material ska samlas in?**

Ange vilken eller vilka typer av biologiskt material som ska samlas in för detta forskningsprojekt. Om alternativet Annat biologiskt material väljs ska dessa anges.

- ☐ Vävnad
- ☐ Blod
- ☒ Annat biologiskt material, ange vad

Serum

14.1.2. [Om ja 14.1] **Kommer det biologiska materialets ursprung gå att härleda till en enskild person, det vill säga går det att spåra eller koppla samman det biologiska materialet med den individ som materialet kommer ifrån?**

Det går att koppla samman det biologiska materialet med individen på två sätt: Antingen direkt (proverna har då märkts med identifierande uppgifter som initialer, födelsenummer eller liknande) eller genom en kodnyckel (materialet är då pseudonymiserat). Om det inte går att koppla samman det biologiska materialet med individen är materialet avidentifierat.

Ja

14.1.2.1. [Om ja 14.1.2] **Ange hur det biologiska materialet kommer att kodas.**

När ett material kodas ersätts individens identitet på materialet med en kod, och en kodnyckel tas fram. Genom kodnyckeln går det att identifiera vilken individ materialet kommer ifrån.

Proverna ges ID-nummer. ID-numret läggs in i databas som upprättas vid Karolinska Universitetslaboratoriet, där behörig personal har möjlighet att knyta det till forskningspersonens identitet.

14.1.3. [Om ja 14.1] **Hur mycket biologiskt material är planerat att samlas in?**

Ange antalet prover, antalet bitar och/eller volym som ska samlas in vid varje enskilt provtagningstillfälle. Ange även det totala antalet prover, antalet bitar och/eller den totala volymen för studien. Ange i förekommande fall även antal prover, bitar och/eller volym för varje delstudie.

Blodprov och Serumprov om 5 milliliter tas dagligen under behandlingperioden som varar 10 dagar.

14.1.4. [Om ja 14.1] **Hur länge ska det biologiska materialet vara tillgängligt för projektet?**

Ange under vilken tid forskningsprojektet kommer ha tillgång till det biologiska materialet.

2020-11-16 – 2030-11-15

14.1.5. [Om ja 14.1] **Kommer det biologiska materialet analyseras och förstöras inom sex månader efter provtagningstillfället, det vill säga är undantagsregeln tillämplig?**

Den 1 januari 2019 infördes en ny undantagsregel gällande prov som tas för forskning men som inte ska sparas i biobank. Undantagsregeln innebär att biobankslagen inte är tillämplig på prov som är avsedda för forskning och som analyseras inom sex månader efter provtagningstillfället och förstörs omedelbart efter analysen. Båda villkoren måste uppfyllas.

Även om undantagsregeln blir tillämplig i aktuellt forskningsprojekt ska alla frågor om biologiskt material besvaras med undantag för frågorna om namn och huvudman för biobanken.

Nej

For peer review only - http://bmjopen.bmj.com/site/about/guidelines.xhtml

14.1.6. [Om ja 14.1] **Ange en motivering till valet av tid som projektet kommer ha tillgång till det biologiska materialet.**

Erfarenheter av tidigare epidemier är att de ofta kommer i flera vågor, ibland under flera år. Det kan därför vara viktigt att kunna ta fram tidigare prov för parallell analys med nytagna prover. Analysmetoderna förändras snabbt och redan om något år är det troligen så stora skillnader att resultat från tidigare analyser inte kan jämföras med resultat från nya analyser på ett tillräckligt pålitligt sätt. Slutligen avser vi att publicera studien och källmaterial från publicerade studier bör vara bevarade i 10 års tid som en säkerhet för att förebygga forskningsfusk.

14.1.7. [Om ja 14.1] **Hur länge ska det biologiska materialet vara tillgängligt efter att projektet är avslutat?**

Ange under vilken tid sökanden planerar att ha tillgång till det biologiska materialet.

2020-11-16 – 2030-11-15

14.1.8. [Om ja 14.1] **Ange en motivering till valet av tid som projektet kommer ha tillgång till det biologiska materialet efter att projektet avslutats.**

Serumprov sparas för eventuell omanalys, dock längst till och med 2030-11-15

14.1.9. [Om ja 14.1] **Ange namnet på den biobank som ska ansvara för provsamlingen.**

Här avses den biobank som kommer att ansvara för den provsamling som bildas under studien, dvs. den samling som det biologiska materialet hämtas till. Ange om möjligt IVO:s registreringsnummer.

Biobank: *En samling biologiskt material från människor, förvarat för ett eller flera ändamål, samt information om detta material. En biobank kan innehålla flera provsamlingar.*

Stockholms Medicinska Biobank

14.1.10. [Om ja 14.1] **Ange huvudman för biobanken**

Ange huvudman för den biobank som ska ansvara för provsamlingen.

Huvudman: den myndighet eller organisation som juridiskt och ekonomiskt har ansvaret för viss verksamhet.

Region Stockholm

14.1.11. [Om ja 14.1] **Vilka analyser kommer att utföras på det biologiska materialet?**

Redogör för alla analyser och bearbetningar som det biologiska materialet ska genomgå.

Kvantitativ PCR och antikroppar mot Corona samt följande kemiska analyser: C-reactive protein (CRP), Procalcitonin, antal vita blodkroppar (WBC), hemoglobin (Hb), Kreatinin, Kreatine Kinas, D-dimer, Troponin, ALT, AST, LD och Bilirubin. Blodprovet efter de kemiska analyserna förstörs, medan serumprovet sparas.

14.1.12. [Om ja 14.1] **Var kommer analyserna att utföras?**

Ange var analyserna på det biologiska materialet kommer att ske. Om analyser ska ske utanför Sverige ska land/länder anges.

Olika regler gäller om prover skickas för analys inom Sverige, inom EU/ESS eller utanför EU/ESS till så kallat tredje land.

☒ I den egna verksamheten
☐ Inom Sverige
☐ Utanför Sverige, ange land/länder

Max 200 tecken

14.1.13. [Om ja 14.1] **Hur kommer det biologiska material att hanteras när analyserna är genomförda?**

Prover i en biobank som har lämnats för analys inom eller utom landet ska lämnas tillbaka till biobank i Sverige eller förstöras (kasseras) när de inte längre behövs för ändamålet. I normalfallet bör det ske inom några få år.

☒ Materialet kommer att förstöras.
☐ Materialet kommer att skickas tillbaka till biobank i Sverige.

For peer review only - http://bmjopen.bmj.com/site/about/guidelines.xhtml

14.2. Planerar projektet att använda biologiskt material från människor från en eller flera befintliga provsamlingar?

Frågan avser om man för detta forskningsprojekt kommer att begära tillgång till biologiskt material eller prover som har samlats in tidigare.

Nej

14.2.1. [Om ja 14.2] Vilket biologiskt material är planerat att användas?

Ange vilken typ av biologiskt material som ska användas i detta forskningsprojekt. Om alternativen "annat biologiskt material" väljs ska dessa anges.

- [ ] Vävnad
- [ ] Blod
- [ ] Annat biologiskt material, ange vad

[Om annat] – Max 100 tecken

14.2.2. [Om ja 14.2] Hur mycket biologiskt material är planerat att användas?

Ange antalet prover, bitar och/eller volymen för allt biologiskt material som är planerat att hämtas in och användas för detta projekt.

Max 2000 tecken

14.2.3. [Om ja 14.2] Ange namnet/namnen på ansvariga biobanker och huvudmän som det biologiska materialet kommer att hämtas ifrån.

Samtliga biobanker och huvudmän som det befintliga biologiska materialet kommer att hämtas eller begäras ut ifrån ska namnges.

Biobank: En samling biologiskt material från människor, förvarat för ett eller flera ändamål samt information om detta material. En biobank kan innehålla flera provsamlingar.

Max 2000 tecken

14.2.4. [Om ja 14.2] Ange namnet på den biobank som kommer att ansvara för denna provsamling.

Här avses den biobank som kommer att ansvara för den nya provsamling som bildas, dvs. den samling som det biologiska materialet hämtas till. Ange om möjligt IVO:s registreringsnummer.

Biobank: En samling biologiskt material på människor, förvarat för ett eller flera ändamål samt information om detta material. En biobank kan innehålla flera provsamlingar.

Max 200 tecken

14.2.5. [Om ja 14.2] Ange huvudman för biobanken.

Ange huvudman för den biobank som ska ansvara för den nya provsamlingen som bildas.

Huvudman: Den myndighet eller organisation som juridiskt och ekonomiskt har ansvaret för viss verksamhet.

Max 200 tecken

14.2.6. [Om ja 14.2] Ange hur materialet är kodat.

När ett material kodas ersätts individens identitet på materialet med en kod, och en kodnyckel tas fram. Genom kodnyckeln går det att identifiera vilken individ materialet kommer ifrån.

Max 2000 tecken

14.2.7. [Om ja 14.2] Vilka analyser kommer att utföras på det biologiska materialet?

Redogör för alla analyser och bearbetningar som det biologiska materialet ska genomgå.

Max 2000 tecken

14.2.8. [Om ja 14.2] Var kommer analyserna att utföras?

Ange var analyserna på det biologiska materialet kommer att ske. Om analyser ska ske utanför Sverige ska land/länder anges.

Olika regler gäller om prover skickas för analys inom Sverige, inom EU/ESS eller utanför EU/ESS till så kallat tredje land.

- [ ] I den egna verksamheten
- [ ] Inom Sverige
- [ ] Utanför Sverige, ange land/länder

For peer review only - http://bmjopen.bmj.com/site/about/guidelines.xhtml

[Ange land/länder] – Max 500 tecken

14.2.9. [Om ja 14.2] **Hur kommer det biologiska materialet att hanteras när analyserna är genomförda?**

Prover i en biobank som har lämnats för analys inom eller utom landet ska lämnas tillbaka till biobank i Sverige eller förstöras (kasseras) när de inte längre behövs för ändamålet. I normalfallet bör det ske inom några få år.

☐ Materialet kommer att förstöras.
☐ Materialet kommer att skickas tillbaka till biobank i Sverige.

14.2.10. [Om ja 14.2] **Hur länge ska det biologiska materialet vara tillgängligt för projektet?**

Ange under vilken tid det biologiska materialet kommer att sparas för det här forskningsprojektet.

Från och med dag månad år – Till och med dag månad år

14.2.11. [Om ja 14.2] **Ange en motivering till valet av tid som det biologiska materialet kommer vara tillgängligt för projektet.**

Max 2000 tecken

14.2.12. [Om ja 14.2] **Hur länge ska det biologiska materialet vara tillgänglig efter att projektet är avslutat?**

Ange under vilken tid sökanden planerar att ha tillgång till det biologiska materialet.

Från och med dag månad år – Till och med dag månad år

14.2.13. [Om ja 14.2] **Ange en motivering till valet av tid som det biologiska materialet ska vara tillgänglig efter att projektet är avslutat.**

Max 2000 tecken

For peer review only

BMJ Open

Convalescent plasma for treatment of COVID-19: Study protocol for an open randomised controlled trial in Sweden

Journal:	*BMJ Open*
Manuscript ID	bmjopen-2020-048337.R2
Article Type:	Protocol
Date Submitted by the Author:	08-Nov-2021
Complete List of Authors:	Dillner, Joakim; Karolinska Institutet Institutionen för laboratoriemedicin; Karolinska University Hospital, Medical Diagnostics Karolinska<br>Ursing, Johan; Danderyd University Hospital, Department of Infectious Diseases
**Primary Subject Heading**:	Infectious diseases
Secondary Subject Heading:	Infectious diseases
Keywords:	COVID-19, Public health < INFECTIOUS DISEASES, Blood bank & transfusion medicine < PATHOLOGY, VIROLOGY, Infection control < INFECTIOUS DISEASES, INTENSIVE & CRITICAL CARE

SCHOLARONE™
Manuscripts

I, the Submitting Author has the right to grant and does grant on behalf of all authors of the Work (as defined in the below author licence), an exclusive licence and/or a non-exclusive licence for contributions from authors who are: i) UK Crown employees; ii) where BMJ has agreed a CC-BY licence shall apply, and/or iii) in accordance with the terms applicable for US Federal Government officers or employees acting as part of their official duties; on a worldwide, perpetual, irrevocable, royalty-free basis to BMJ Publishing Group Ltd ("BMJ") its licensees and where the relevant Journal is co-owned by BMJ to the co-owners of the Journal, to publish the Work in this journal and any other BMJ products and to exploit all rights, as set out in our licence.

The Submitting Author accepts and understands that any supply made under these terms is made by BMJ to the Submitting Author unless you are acting as an employee on behalf of your employer or a postgraduate student of an affiliated institution which is paying any applicable article publishing charge ("APC") for Open Access articles. Where the Submitting Author wishes to make the Work available on an Open Access basis (and intends to pay the relevant APC), the terms of reuse of such Open Access shall be governed by a Creative Commons licence – details of these licences and which Creative Commons licence will apply to this Work are set out in our licence referred to above.

Other than as permitted in any relevant BMJ Author's Self Archiving Policies, I confirm this Work has not been accepted for publication elsewhere, is not being considered for publication elsewhere and does not duplicate material already published. I confirm all authors consent to publication of this Work and authorise the granting of this licence.

For peer review only

Convalescent plasma for treatment of COVID-19: Study protocol for an open randomised controlled trial in Sweden

Joakim Dillner *Professor*[1], Johan Ursing *MD*[2]

1) Center for Cervical Cancer Prevention, Karolinska University Laboratory, Karolinska University Hospital and Department of Laboratory Medicine, Karolinska Institutet, Stockholm, Sweden
2) Department of Infectious Diseases, Danderyd Hospital, Stockholm, Sweden

Correspondence to: Joakim Dillner, Center for Cervical Cancer Prevention, Karolinska University Laboratory, Karolinska University Hospital and Department of Laboratory Medicine, Karolinska Institutet, S-141 86 Stockholm, Sweden

Phone: +46 72 468 24 60; E-mail: Joakim.dillner@ki.se or joakim.dillner@sll.se

Keywords: COVID-19, Sars-CoV-2, convalescent plasma

Word count: 2752

For peer review only - http://bmjopen.bmj.com/site/about/guidelines.xhtml

ABSTRACT

Introduction: Although there are many studies on the use of convalescent plasma (CP) for treatment of COVID-19, it is not clear i) which groups of patients may benefit, ii) what dose of plasma to give or iii) which antibody levels the plasma should contain. Previous phaseI/II studies and literature review suggest that CP should only be given to patients with viremia, that a daily infusion should be given until the patient becomes virus-free and that the neutralizing antibody titer should preferably be >1:640

Methods and analysis: An open randomised controlled trial enrolling patients with COVID-19, who must be SARS-CoV-2 positive in both airway and blood samples and admitted to a study hospital. Block randomization 2:1 is to either 200 ml convalescent plasma (preferably titre ≥1/640) daily for up to 10 days (until virus-negative in blood) plus standard care or standard care only (control arm). The primary endpoint is mortality by day 28 after study inclusion. Secondary endpoints include mortality by day 60 and doses of plasma needed to clear viremia. Assuming a reduced mortality of approximately 30% by the CP therapy and 85-88% survival in the control arm, approximately 600 participants will be enrolled to the CP therapy arm and 300 participants to the control arm.

Ethics and dissemination: Ethical approval has been granted by the Swedish Ethical Review Authority (ref 2020-06277). Results from this trial will be compiled in a clinical study report, disseminated via journal articles and communicated to stakeholders.

Trial registration number: ClinicalTrials.gov NCT04649879

For peer review only - http://bmjopen.bmj.com/site/about/guidelines.xhtml

ARTICLE SUMMARY

Strengths and limitations of this study

- A randomized trial design based on phase I and II studies that first investigated which patients to enroll (viremic patients) as well as how plasma should be characterized (high neutralizing antibody titer) and how it should be given (daily until viral clearance).
- Powered to detect a 30% mortality decrease.
- Clearance of viremia is a secondary endpoint that will be very rapid to evaluate
- Availability of high-titer convalescent plasma blood group matched to study participants may be a limiting factor
- This is an unblinded trial

For peer review only

INTRODUCTION

Corona virus disease 2019 (COVID-19) is an ongoing pandemic caused by the severe acute respiratory syndrome coronavirus 2 (SARS-CoV-2). Current treatment options are limited. Corticosteroid therapy (dexamethasone, hydrocortisone or methylprednisolone) for up to 10 days has been shown to significantly reduce mortality in severely ill COVID-19 patients, summary odds ratio, 0.66 (95% CI 0.53-0.82).[1,2] Dexamethasone also reduced the risk of progression to invasive mechanical ventilation (RR 0.76 (0.61-0.96) p=0.021). However, no improvement was seen in patients not requiring supplemental oxygen therapy and corticosteroids were associated with prolonged viral shedding.[1,3,4]

Convalescent plasma (CP) therapy is a form of immunotherapy that has been used for the treatment and prevention of infectious diseases for more than 100 years. CP was successfully used in the treatment of SARS, MERS and the 2019 H1N1 pandemic.[5-8] A meta-analysis of 32 SARS and severe influenza studies showed that CP treatment was associated with a significant reduction in the pooled odds of mortality (odds ratio, 0.25; 95% confidence interval, 0.14-0.45).[9] However, CP treatment did not significantly improve the survival of patients with Ebola disease.[10] This was possibly due to a lack of data on neutralizing antibody titres.

Early studies on CP therapy suggested that in COVID-19, CP should be given daily until the patient becomes virus-free.[11-14] In America more than 70 000 patients have received CP. A safety report on 20 000 patients indicated that CP was well tolerated and not associated with severe adverse events.[15] In a cohort of 1000 patients, mortality was significantly lower in patients receiving CP with antibody titres ≥1/640 compared to patients receiving CP with lower titres if the treatment was initiated within 72 hours of admission (data available at www.fda.gov). Though the numbers treated are large and the results suggest a beneficial

For peer review only - http://bmjopen.bmj.com/site/about/guidelines.xhtml

effect, the lack of controls leaves space for much uncertainty. Moreover, there are randomised studies reporting no beneficial effect, although CP therapy was not specifically given to viremic patients in these trials.[16-18] There is thus a need for a randomised clinical trial that focusses on a population at high risk of severe COVID-19 (i.e. viremic patients in early disease) and on which plasma to give and how (preferably neutralizing antibodies of at least 1:640 and one plasma bag infused daily until clearance of viremia occurs).

We found that viremia upon admission identifies patients at 7-fold increased risk of admission to intensive care and 8-fold increased risk of death.[19] CP treatment appeared to result in rapid viral clearance in a small case series and two randomized trials.[16,18,20] CP appeared to be well tolerated in a phase I study in which patients only received one dose of CP and a phase II study in which CP was given until viremia disappeared (JU, unpublished data). Moreover, mortality was 0/6, 0/18, and 6/10 in patients that were not viremic when treated, patients that were treated until viremia disappeared and patients in whom viremia continued despite treatment, respectively. The last group consisted of patients in whom treatment was stopped due to adverse events, lack of CP or because they were part of the initial phase I study in which only one dose was given. Viremic patients may thus benefit from CP and can be identified early prior to development of severe COVID-19.

Rationale

Convalescent plasma has been shown to be safe and effective for treatment of several diseases. Data indicate that it is safe for treatment of COVID-19. Preliminary data indicate that treatment should be given early prior to development of severe illness. A group at high risk of severe disease and death that has the most to benefit from CP can be easily identified by detection of viremia upon admission.[19] Phase II study data indicates that treatment should be given until SARS-CoV-2 is no longer detected in serum and the donor antibody

For peer review only - http://bmjopen.bmj.com/site/about/guidelines.xhtml

neutralization titres should be ≥1/640. A randomised controlled trial in which viremic patients are treated with CP with the equivalent of an antibody titre ≥1/640 is thus required to determine if CP can be an effective COVID-19 treatment.

Objective

The aim is to assess the efficacy, safety and tolerability of convalescent plasma for treatment of patients with COVID-19 and viremia.

For peer review only

For peer review only - http://bmjopen.bmj.com/site/about/guidelines.xhtml

METHODS AND ANALYSIS

Study design

A multicentre, open 2:1 randomised controlled clinical trial conducted in Sweden. Study sites include Karolinska University Hospital, Danderyd Hospital and the Hospital in Falun (for a complete list of study sites, visit clinicaltrials.gov NCT04649879). All Hospitals with infectious Diseases departments in Sweden have been invited to participate. The study will be conducted in accordance with the Helsinki declaration on ethical principles for medical research involving human subjects. The study is expected to take place during 2021 (2020-12-03 – 2022-02-01). This study protocol is prepared using the SPIRIT guidelines.[21]

Participants

Patients with COVID-19 admitted to a study hospital will be considered for study entry. Patients will be identified by notification amongst doctors at the hospitals. Patients will be informed of the study and offered to participate if they match inclusion and exclusion criteria. An electronic (REDCap) case record form will be filled in for all screened and included patients. Upon inclusion an informed consent form will be signed. Enrolment of patients is done by dedicated physicians and expected to take place during a 12-month period. Intervention is taking place up to a maximum of 10 consecutive days and follow up will be on day 28 and day 60 after inclusion (Table 1). Adherence to study protocol will be monitored by registry linkages with the patient registry.

Eligibility criteria

Inclusion criteria for participants:

1. Age ≥18

For peer review only - http://bmjopen.bmj.com/site/about/guidelines.xhtml

2. Admitted to a study hospital

3. Active COVID-19 defined as symptoms + SARS CoV-2 identified from upper or lower airway samples and blood

4. Negative pregnancy test taken before inclusion and use of an acceptable effective method of contraception until treatment discontinuation if the participant is a woman of childbearing potential

5. Written informed consent after meeting with a study physician and ability and willingness to complete follow up

Exclusion criteria for participants:

1. No matching plasma donor (Exact matching in the ABO system is required)

2. Unavailability of plasma

3. Estimated glomerular filtration rate <30 (kidney failure stage III or more)

4. Pregnancy (urinary-hcg)

5. Breast feeding

6. Inability to give informed consent

The study will be conducted at wards where COVID-19 patients are treated in study hospitals. To be eligible to participate, personnel involved in the study will receive appropriate training relevant to the study.

Intervention

For peer review only - http://bmjopen.bmj.com/site/about/guidelines.xhtml

Patients will be randomised 2:1 to treatment with convalescent plasma plus standard care or standard care only. Randomisation is by random permutated blocks using REDCap or equivalent.

- Participants will receive 200 ml convalescent plasma daily until SARS-CoV-2 is no longer detectable in the blood up to a maximum of 10 CP infusions. CP will be given as a slow infusion over 2 hours. CP neutralization titre of ≥ 1/640 (which is higher than conventionally used as high titer plasma) or an ELISA reactivity against the Spike protein of SARS-CoV-2 by the Euroimmun commercial assay >9 is desired. The in-house neutralization test is performed as described.[22]

New antibody tests are under development and can be used instead if equivalence to neutralization or Euroimmun ELISA is demonstrated. If no high titre plasma is available, lower antibody titre plasma may be given but must in no instance be lower than 4 in the Euroimmun ELISA. CP will be obtained from respective study sites blood bank. If this is not possible, CP will be available from the Department of clinical immunology and transfusion medicine Karolinska University Hospital.

Patients will be monitored for adverse events, especially allergic reactions and impaired renal function. Patients not able to receive study treatment will receive standard care.

If an attending physician assesses that a patient is suffering from an adverse event induced by the plasma infusion and requiring treatment, the infusion will be stopped for that patient. The outcome will be classified as an adverse event necessitating treatment stop.

A study subject can terminate his/her participation in the study at any time without giving a reason why. Should a subject wish to do so he/she will be treated in line with standard recommendations.

Study organisers can end a patient's participation for safety reasons.

For peer review only - http://bmjopen.bmj.com/site/about/guidelines.xhtml

Outcomes

Primary endpoint: mortality by day 28 after inclusion into the study.

Secondary endpoints:

1. Mortality by day 60 after inclusion into the study

2. Requirement of invasive ventilation or $Pao_2/FiO_2 \leq 70$ for ≥ 12 hours in the case of patients not eligible for intensive care

3. Adverse events

4. Dose of plasma needed to clear viremia

5. Time to clearance of viremia

Sample size

Available data indicates that CP is safe and that high titre CP can reduce mortality by approximately 30%. For that reason, a 2:1 randomisation is preferred. Randomization will be done by computer-generated random numbers. Based on available data we assume 85-88% survival in the control arm. Assuming a power of 80% and an alpha of 0,05%, 920 patients need to be enrolled.

Recruitment

To reach the number of patients needed to conduct the study with enough power, all hospitals in Sweden with patients acutely ill in COVID-19 are invited to participate.

For peer review only - http://bmjopen.bmj.com/site/about/guidelines.xhtml

Measurements

Blood samples for analysis of the following routine blood chemistry will be taken prior to start of treatment if available at respective study sites: C-reactive protein (CRP), Procalcitonin, white blood cell count (WBC), haemoglobin (Hb), Creatinine, Creatine Kinase, D-dimer, troponin, ALT, AST, LD, and bilirubin.

Blood samples for detection of SARS-CoV-2 in the blood will be taken prior to treatment start, daily during treatment and until two consecutive negative results are obtained. Serological samples will be collected for subsequent SARS-CoV-2 culture whenever PCR samples are taken.

Data collection

The following basic data will be collected and entered into electronic records (REDCap):

- Age, gender and reason for non-inclusion for screened but not included patients
- Date of symtom onset and date of start of CP theraphy will be recorded and used in subgroup analyses.
- Basic patient characteristics, comorbidities, disease severity characteristics and treatment upon study entry.
- Each dose of CP and date it was given along with any observed adverse events
- Concomitant treatment with antivirals and / or corticosteroids
- The outcome of daily SARS-CoV-2 PCR until 2 consecutive negative PCRs are recorded.
- The need for mechanical ventilation and date when this was initiated

For peer review only - http://bmjopen.bmj.com/site/about/guidelines.xhtml

- For patients not eligible for intensive care: each day when PaO2/FiO2 ratio was less than 70 for ≥ 12 hours. PaO2 and FiO2 will be estimated from SO2% and O2 flow in nasal cannula, face mask or face mask with reservoir based on EPIC II data. A ratio of 70 is approximately equal to 90% SO2 with 8-9 L of Oxygen flow using a face mask with a reservoir.
- Date of death
- Survival or death by day 28 and day 60

Data is entered directly into a computerised system that includes automatic range checks.

Data analysis plan

Efficacy outcomes will be determined using cox regressions analyses, non-parametric regression analyses and survival analyses of the cohort of patients that fulfilled entry criteria but not exclusion criteria. A per protocol and intention to treat analysis will be done. Final analysis will include a description of included participants, proportions of adverse events and any serious adverse events, the proportion of participants withdrawn or lost to follow up, the cumulative success and failure rates by day 28 and day 60. Categorical variables will be compared using the $\chi 2$ test or Fisher exact test and continuous variables using quantile regression. The final data analysis will be performed when a total number of 88 outcome events have occurred. An interim analysis will be performed after 44 outcome events.

Patient and Public Involvement statement

No patient or patient organization was involved in design, recruitment or management of the study.

ETHICS AND DISSEMINATION

For peer review only - http://bmjopen.bmj.com/site/about/guidelines.xhtml

The trial has been registered with clinicaltrials.gov NCT04649879, and sponsor protocol number CP03. The investigational product is a blood product (not classified as a drug) and the regulatory agency is the National Inspection Authority for Health and Care (IVO), that has approved the trial. The trial has been approved by the National Ethical Review Agency (decision 2020-06277). Patients will be informed of the study verbally and in writing. Details about the trial including risks and benefits will be explained and any questions answered, including the information that all participants in the study are covered by the regular patient injury insurance. A copy of the written consent form will then be signed. If an amendment substantially alters the study design, or increases the potential risk to the patients, written informed consent must be obtained again for currently enrolled patients and must be provided to additional patients prior to their entry into the study.

The trial will be conducted in compliance with the ICH guidelines for Good Clinical Practice, the latest edition of the Declaration of Helsinki and any local regulations.

Upon inclusion an entry will be made in the patient's medical record specifying that the subject is participating in this study, if convalescent plasma treatment is given, and that written informed consent has been obtained. Any apparent side effects experienced by the subject will be assessed from the time the subject signs the informed consent and as long as the subject is part of the study, and will be reported by study site personnel either as a Baseline Event or an Adverse Event (AE). The study personnel will document any Baseline Events or AEs in the eCRF, whether observed by the investigator or reported by the subject. The safety of the different treatment arms will be assessed with regard to AEs, baseline medical conditions, and findings from the physical examination and laboratory tests. All AEs, irrespective of nature, will be followed until resolution or until end of the follow-up period.

For peer review only - http://bmjopen.bmj.com/site/about/guidelines.xhtml

The reporting period for AEs starts at inclusion and ends at the final follow-up visit 2 months after inclusion. Interim analyses will be done after inclusion of 100, 300 and 600 patients. The study will be stopped if a 30% difference in treatment outcome is detected and an Independent Data Monitoring Committee will be convened to review the data and determine if the study should be stopped or not. An external party will monitor the study regularly and ensure that the study follows GCP. Audits or inspections will be performed at the study site during or after the study. These visits may include source data verification and confidentiality documents are therefore created.

After completion of the study, a clinical study report will be prepared that will form the basis for a manuscript intended for publication in a medical journal. Data will only be reported in aggregated form, without being able to be attributed to an individual. Records of the study will be kept for 10 years after final signing of the Clinical Study Report. Data will be entered into a database by the investigators at the coordinating hospital (Danderyd Hospital). Study ID only will be used on computerised records. The key will be stored with the CRFs at Danderyd Hospital. Only the sponsor, principal investigator and participating researchers will have access to all data. Source data comprising electronic medical records will be stored at each study site.

Data sharing statement

The data constitutes sensitive data about health of human research subjects. However, pseudonymised, individual-level data that allow full replication of the results in this article will be made freely available from joakim.dillner@sll.se. The study protocol is available at clinicaltrials.gov NCT04649879.

For peer review only

For peer review only - http://bmjopen.bmj.com/site/about/guidelines.xhtml

Author Contributions

JD and JU conceived the study and wrote the paper. Both authors approved the final version of the manuscript.

Funding

This work was supported by the Swedish Research Council (2020-05793_3). The funding agency has had no role in the design, execution or interpretation of the study or in the decision to submit for publication.

Competing interests

None declared.

For peer review only

REFERENCES

1. Horby P, Lim WS, Emberson JR, et al. Dexamethasone in Hospitalized Patients with Covid-19 - Preliminary Report. *N Engl J Med* 2020 Jul 17:NEJMoa2021436.

2. Sterne JAC, Murthy S, Diaz JV, et al. Association Between Administration of Systemic Corticosteroids and Mortality Among Critically Ill Patients With COVID-19: A Meta-analysis. *JAMA* 2020; 324:1330-1341.

3. Arabi YM, Mandourah Y, Al-Hameed F, et al. Corticosteroid Therapy for Critically Ill Patients with Middle East Respiratory Syndrome. *Am J Respir Crit Care Med* 2018;197:757-67.

4. Stockman LJ, Bellamy R, Garner P. SARS: systematic review of treatment effects. *PLoS Med* 2006;3:e343.

5. Hung IF, To KK, Lee CK, et al. Convalescent plasma treatment reduced mortality in patients with severe pandemic influenza A (H1N1) 2009 virus infection. *Clin Infect Dis* 2011;52:447-56.

6. Zhou B, Zhong N, Guan Y. Treatment with convalescent plasma for influenza A (H5N1) infection. *N Engl J Med* 2007;357:1450-1.

7. Cheng Y, Wong R, Soo YO, et al. Use of convalescent plasma therapy in SARS patients in Hong Kong. *Eur J Clin Microbiol Infect Dis* 2005;24:44-6.

8. Ko JH, Seok H, Cho SY, et al. Challenges of convalescent plasma infusion therapy in Middle East respiratory coronavirus infection: a single centre experience. *Antivir Ther* 2018;23:617-22.

For peer review only - http://bmjopen.bmj.com/site/about/guidelines.xhtml

9. Mair-Jenkins J, Saavedra-Campos M, Baillie JK, et al. The effectiveness of convalescent plasma and hyperimmune immunoglobulin for the treatment of severe acute respiratory infections of viral etiology: a systematic review and exploratory meta-analysis. *J Infect Dis* 2015;211:80-90.

10. van Griensven J, Edwards T, de Lamballerie X, et al. Evaluation of Convalescent Plasma for Ebola Virus Disease in Guinea. *N Engl J Med* 2016;374:33-42.

11. Ye M, Fu D, Ren Y, et al. Treatment with convalescent plasma for COVID-19 patients in Wuhan, China. *J Med Virol* 2020;92:1890-1901.

12. Ahn JY, Sohn Y, Lee SH, et al. Use of Convalescent Plasma Therapy in Two COVID-19 Patients with Acute Respiratory Distress Syndrome in Korea. *J Korean Med Sci* 2020;35:e149.

13. Duan K, Liu B, Li C, et al. Effectiveness of convalescent plasma therapy in severe COVID-19 patients. *Proc Natl Acad Sci U S A* 2020;117:9490-9496.

14. Shen C, Wang Z, Zhao F, et al. Treatment of 5 Critically Ill Patients With COVID-19 With Convalescent Plasma. *JAMA* 2020;323:1582-1589.

15. Joyner MJ, Bruno KA, Klassen SA, et al. Safety Update: COVID-19 Convalescent Plasma in 20,000 Hospitalized Patients. *Mayo Clin Proc* 2020;95:1888-97.

16. Li L, Zhang W, Hu Y, et al. Effect of Convalescent Plasma Therapy on Time to Clinical Improvement in Patients With Severe and Life-threatening COVID-19: A Randomized Clinical Trial. *JAMA* 2020;324:460-470.

17. Simonovich VA, Burgos Pratx LD, et al. A Randomized Trial of Convalescent Plasma in Covid-19 Severe Pneumonia. *N Engl J Med* 2020 Nov 24. doi: 10.1056/NEJMoa2031304.

For peer review only

For peer review only - http://bmjopen.bmj.com/site/about/guidelines.xhtml

18. Agarwal A, Mukherjee A, Kumar G, et al. Convalescent plasma in the management of moderate covid-19 in adults in India: open label phase II multicentre randomised controlled trial (PLACID Trial) *BMJ* 2020;371:m3939.

19. Hagman K, Hedenstierna M, Gille-Johnson P, et al. SARS-CoV-2 RNA in serum as predictor of severe outcome in COVID-19: a retrospective cohort study. *Clin Infect Dis* 2020. doi: 10.1093/cid/ciaa1285

20. Zeng QL, Yu ZJ, Gou JJ, et al. Effect of Convalescent Plasma Therapy on Viral Shedding and Survival in Patients With Coronavirus Disease 2019. *J Infect Dis* 2020;222:38-43.

21. Chan A-W, Tetzlaff JM, Gøtzsche PC, et al. SPIRIT 2013 Explanation and Elaboration: Guidance for protocols of clinical trials. *BMJ* 2013;346:e7586.

22. Varnaitė R, García M, Glans H, et al. Expansion of SARS-CoV-2-Specific Antibody-Secreting Cells and Generation of Neutralizing Antibodies in Hospitalized COVID-19 Patients. *J Immunol.* 2020;205:2437-2446.

For peer review only

For peer review only - http://bmjopen.bmj.com/site/about/guidelines.xhtml

Table 1. Planned questionnaire and sampling follow up

Time point (days)	Prior to or upon inclusion	Daily during treatment	28	60
**Informed Consent**	X			
**CRF**	X	**X**	**X**	**X**
**Routine blood chemistry**	X			
**Serum sample**	X	X		
**Virus PCR in blood**	X	X		

For peer review only

Reporting checklist for protocol of a clinical trial.

Based on the SPIRIT guidelines.

Instructions to authors

Complete this checklist by entering the page numbers from your manuscript where readers will find each of the items listed below.

Your article may not currently address all the items on the checklist. Please modify your text to include the missing information. If you are certain that an item does not apply, please write "n/a" and provide a short explanation.

Upload your completed checklist as an extra file when you submit to a journal.

In your methods section, say that you used the SPIRITreporting guidelines, and cite them as:

Chan A-W, Tetzlaff JM, Gøtzsche PC, Altman DG, Mann H, Berlin J, Dickersin K, Hróbjartsson A, Schulz KF, Parulekar WR, Krleža-Jerić K, Laupacis A, Moher D. SPIRIT 2013 Explanation and Elaboration: Guidance for protocols of clinical trials. BMJ. 2013;346:e7586

		Reporting Item	Page Number
**Administrative information**			
Title	#1	Descriptive title identifying the study design, population, interventions, and, if applicable, trial acronym	1
Trial registration	#2a	Trial identifier and registry name. If not yet registered, name of intended registry	2
Trial registration: data set	#2b	All items from the World Health Organization Trial Registration Data Set	1-15
Protocol version	#3	Date and version identifier	2020-12-21 version 1.2
Funding	#4	Sources and types of financial, material, and other support	15
Roles and responsibilities: contributorship	#5a	Names, affiliations, and roles of protocol contributors	1, 15

For peer review only

Roles and responsibilities: sponsor contact information	#5b	Name and contact information for the trial sponsor	1
Roles and responsibilities: sponsor and funder	#5c	Role of study sponsor and funders, if any, in study design; collection, management, analysis, and interpretation of data; writing of the report; and the decision to submit the report for publication, including whether they will have ultimate authority over any of these activities	15
Roles and responsibilities: committees	#5d	Composition, roles, and responsibilities of the coordinating centre, steering committee, endpoint adjudication committee, data management team, and other individuals or groups overseeing the trial, if applicable (see Item 21a for data monitoring committee)	14
**Introduction**			
Background and rationale	#6a	Description of research question and justification for undertaking the trial, including summary of relevant studies (published and unpublished) examining benefits and harms for each intervention	4-6
Background and rationale: choice of comparators	#6b	Explanation for choice of comparators	10
Objectives	#7	Specific objectives or hypotheses	6
Trial design	#8	Description of trial design including type of trial (eg, parallel group, crossover, factorial, single group), allocation ratio, and framework (eg, superiority, equivalence, non-inferiority, exploratory)	7
**Methods: Participants,**			

For peer review only

**interventions, and outcomes**			
Study setting	#9	Description of study settings (eg, community clinic, academic hospital) and list of countries where data will be collected. Reference to where list of study sites can be obtained	7
Eligibility criteria	#10	Inclusion and exclusion criteria for participants. If applicable, eligibility criteria for study centres and individuals who will perform the interventions (eg, surgeons, psychotherapists)	7-8
Interventions: description	#11a	Interventions for each group with sufficient detail to allow replication, including how and when they will be administered	9
Interventions: modifications	#11b	Criteria for discontinuing or modifying allocated interventions for a given trial participant (eg, drug dose change in response to harms, participant request, or improving / worsening disease)	10
Interventions: adherance	#11c	Strategies to improve adherence to intervention protocols, and any procedures for monitoring adherence (eg, drug tablet return; laboratory tests)	7
Interventions: concomitant care	#11d	Relevant concomitant care and interventions that are permitted or prohibited during the trial	8-9
Outcomes	#12	Primary, secondary, and other outcomes, including the specific measurement variable (eg, systolic blood pressure), analysis metric (eg, change from baseline, final value, time to event), method of aggregation (eg, median, proportion), and time point for each outcome. Explanation of the clinical relevance of chosen efficacy and harm outcomes is strongly recommended	10

For peer review only

Participant timeline	#13	Time schedule of enrolment, interventions (including any run-ins and washouts), assessments, and visits for participants. A schematic diagram is highly recommended (see Figure)	19
Sample size	#14	Estimated number of participants needed to achieve study objectives and how it was determined, including clinical and statistical assumptions supporting any sample size calculations	10
Recruitment	#15	Strategies for achieving adequate participant enrolment to reach target sample size	7
**Methods: Assignment of interventions (for controlled trials)**			
Allocation: sequence generation	#16a	Method of generating the allocation sequence (eg, computer-generated random numbers), and list of any factors for stratification. To reduce predictability of a random sequence, details of any planned restriction (eg, blocking) should be provided in a separate document that is unavailable to those who enrol participants or assign interventions	9
Allocation concealment mechanism	#16b	Mechanism of implementing the allocation sequence (eg, central telephone; sequentially numbered, opaque, sealed envelopes), describing any steps to conceal the sequence until interventions are assigned	9
Allocation: implementation	#16c	Who will generate the allocation sequence, who will enrol participants, and who will assign participants to interventions	7,9

For peer review only

Blinding (masking)	#17a	Who will be blinded after assignment to interventions (eg, trial participants, care providers, outcome assessors, data analysts), and how	N/A
Blinding (masking): emergency unblinding	#17b	If blinded, circumstances under which unblinding is permissible, and procedure for revealing a participant's allocated intervention during the trial	N/A
**Methods: Data collection, management, and analysis**			
Data collection plan	#18a	Plans for assessment and collection of outcome, baseline, and other trial data, including any related processes to promote data quality (eg, duplicate measurements, training of assessors) and a description of study instruments (eg, questionnaires, laboratory tests) along with their reliability and validity, if known. Reference to where data collection forms can be found, if not in the protocol	11-12
Data collection plan: retention	#18b	Plans to promote participant retention and complete follow-up, including list of any outcome data to be collected for participants who discontinue or deviate from intervention protocols	19
Data management	#19	Plans for data entry, coding, security, and storage, including any related processes to promote data quality (eg, double data entry; range checks for data values). Reference to where details of data management procedures can be found, if not in the protocol	12
Statistics: outcomes	#20a	Statistical methods for analysing primary and secondary outcomes. Reference to	12

For peer review only

		where other details of the statistical analysis plan can be found, if not in the protocol	
Statistics: additional analyses	#20b	Methods for any additional analyses (eg, subgroup and adjusted analyses)	12
Statistics: analysis population and missing data	#20c	Definition of analysis population relating to protocol non-adherence (eg, as randomised analysis), and any statistical methods to handle missing data (eg, multiple imputation)	12
**Methods: Monitoring**			
Data monitoring: formal committee	#21a	Composition of data monitoring committee (DMC); summary of its role and reporting structure; statement of whether it is independent from the sponsor and competing interests; and reference to where further details about its charter can be found, if not in the protocol. Alternatively, an explanation of why a DMC is not needed	14
Data monitoring: interim analysis	#21b	Description of any interim analyses and stopping guidelines, including who will have access to these interim results and make the final decision to terminate the trial	14
Harms	#22	Plans for collecting, assessing, reporting, and managing solicited and spontaneously reported adverse events and other unintended effects of trial interventions or trial conduct	13
Auditing	#23	Frequency and procedures for auditing trial conduct, if any, and whether the process will be independent from investigators and the sponsor	14
**Ethics and dissemination**			

For peer review only

Research ethics approval	#24	Plans for seeking research ethics committee / institutional review board (REC / IRB) approval	2<br><br>(Approval obtained 2020-12-02 from the Swedish Ethical Review Authority (ref 2020-06277).)
Protocol amendments	#25	Plans for communicating important protocol modifications (eg, changes to eligibility criteria, outcomes, analyses) to relevant parties (eg, investigators, REC / IRBs, trial participants, trial registries, journals, regulators)	13
Consent or assent	#26a	Who will obtain informed consent or assent from potential trial participants or authorised surrogates, and how (see Item 32)	7
Consent or assent: ancillary studies	#26b	Additional consent provisions for collection and use of participant data and biological specimens in ancillary studies, if applicable	N/A
Confidentiality	#27	How personal information about potential and enrolled participants will be collected, shared, and maintained in order to protect confidentiality before, during, and after the trial	14
Declaration of interests	#28	Financial and other competing interests for principal investigators for the overall trial and each study site	15
Data access	#29	Statement of who will have access to the final trial dataset, and disclosure of contractual agreements that limit such access for investigators	14
Ancillary and post trial care	#30	Provisions, if any, for ancillary and post-trial care, and for compensation to those who suffer harm from trial participation	13

For peer review only

Dissemination policy: trial results	#31a	Plans for investigators and sponsor to communicate trial results to participants, healthcare professionals, the public, and other relevant groups (eg, via publication, reporting in results databases, or other data sharing arrangements), including any publication restrictions	14
Dissemination policy: authorship	#31b	Authorship eligibility guidelines and any intended use of professional writers	N/A
Dissemination policy: reproducible research	#31c	Plans, if any, for granting public access to the full protocol, participant-level dataset, and statistical code	14
**Appendices**			
Informed consent materials	#32	Model consent form and other related documentation given to participants and authorised surrogates	Appendix 1. Study participant's information and consent form (as approved by the Swedish Ethical Review Authority (ref 2020-06277).)
Biological specimens	#33	Plans for collection, laboratory evaluation, and storage of biological specimens for genetic or molecular analysis in the current trial and for future use in ancillary studies, if applicable	Appendix 2. Information on biological specimens (as approved by the Swedish Ethical Review Authority (ref 2020-06277).)

None The SPIRIT checklist is distributed under the terms of the Creative Commons Attribution License CC-BY-ND 3.0. This checklist can be completed online using https://www.goodreports.org/, a tool made by the EQUATOR Network in collaboration with Penelope.ai

For peer review only

APPENDIX 1

Forskningspersonsinformation och informerat samtycke

Avseende studien ”Konvalescentplasma för behandling av COVID-19: en randomiserad kontrollerad studie”

Du tillfrågas härmed om att delta i en studie där plasma från personer som tillfrisknat från en SARS-CoV-2 infektion ges som tillägg till standard-behandling vid behandling av COVID-19.

Det är helt frivilligt att delta i studien och om du väljer att inte delta påverkar det inte din fortsatta behandling.

1. Bakgrund och syfte

Infektionssjukdomen COVID-19 ofta kallat corona orsakas av viruset SARS-CoV-2. Viruset orsakar allt från lindriga förkylningssymptom till en virusorsakad lunginflammation och död. Förekomst av virus i blod är en tidig markör för allvarlig sjukdom med ökad risk för död.

Konvalescentplasma är en behandling som används eller är under utvärdering i många länder. Plasma är den del av blodet som blir kvar när man tar bort de röda och vita blodkropparna. Plasma innehåller antikroppar som är en del av kroppens immunförsvar mot en infektion. Plasma från personer som haft COVID-19 (konvalescentplasma) innehåller höga nivåer av antikroppar riktade mot SARS-CoV-2. I USA har fler än 70 000 personer behandlats med sådan plasma. Behandlingen har visats vara väl tolererad och minskar möjligen risken för död. Behandlingen har därför godkänts av den Amerikanska motsvarigheten till läkemedelsverket. Om plasma verkligen gör nytta är dock inte säkerställt. Vidare är den optimala dosen oklar liksom när i sjukdomsförloppet det bör ges.

Syftet med denna studie är att utvärdera om konvalescentplasma minskar risken för död orsakad av COVID-19 när det ges till personer med virus i blod.

2. Förfrågan om deltagande

Du tillfrågas härmed om du vill delta i en denna studie där plasma från personer som har antikroppar mot SARS-CoV-2 ges till personer med akut COVID-19 sjukdom. Patienter som behandlas för coronainfektion vid Karolinska Universitetssjukhuset, Danderyds sjukhus, Södersjukhuset, Mälarsjukhuset i Eskilstuna, Gävle sjukhus, Centralsjukhuset i Karlstad eller Falu lasarett får erbjudande att delta i studien. Ansvarig för studien är Karolinska Universitetssjukhuset.

3. Hur går studien till?

Du lottas via ett datorprogram till standardbehandling eller konvalescensplasma i tillägg till standardbehandling. 2/3 av inkluderade forskningspersoner lottas till konvalescentplasma och 1/3 till standardbehandling. Cirka 20 ml blod tas vid behandlingsstart för att mäta nivån av inflammation i kroppen och identifiera din blodgrupp. Forskningspersoner som lottas till behandling får en dos konvalescensplasma à 200 ml dagligen tills virus inte längre påvisas i blod eller maximalt 10 dagar. Tio ml blod tas dagligen för att kontrollera om du har virus i blod. Uppföljning via genomgång av din journal sker dag 28 och dag 60. Den maximala totala mängden blod som tas för studien är 120 ml. Mängden blod påverkar inte din hälsa negativt. Blodprovet som tas för viruspåvisning sparas för eventuella utvidgade mikrobiella och immunologiska analyser.

For peer review only

5. Vilka är riskerna?

Plasmatransfusion är en välbeprövad klinisk rutin vid ett flertal olika sjukdomstillstånd inklusive COVID-19. Potentiella biverkningar är feber, huvudvärk, svimning eller allergisk reaktion. Andra kända biverkningar som plasma kan orsaka är övervätskning och i mycket sällsynta fall en sorts lungskada som orsakas av en specifik sorts antikropp. Konvalescentplasman som ges testas för dessa antikroppar för att säkerställa att lungskada inte uppkommer och den dagliga mängden plasma är liten, för att undvika övervätskning.

6. Finns det några fördelar?

Tidigare studier visar att konvalescensplasma möjligen minskar risken för död orsakad av COVID-19.

7. Hantering av data och sekretess

Alla data hanteras i enlighet med dataskyddsförordningen (GDPR) och sparas i 10 år. Den rättsliga grunden för detta är ditt samtycke. Du har rätt att när som helst ta tillbaka ditt samtycke.

Kliniska data kommer att pseudonymiseras genom att varje individ får en unik kod (studienummer) som länkas till personnumret. Koden som kopplar samman studienummer och personnummer kommer att förvaras i ett låst skåp i ett låst rum på Danderyds sjukhus. Kodad studiedata kommer att överföras till en studiedatabas. Dina uppgifter kommer att behandlas för statistiska beräkningar och när resultaten publiceras i medicinsk facklitteratur kommer den enskilda individen inte att kunna identifieras.

För att säkerställa att de uppgifter som samlas in och registreras är korrekta kan dessa komma att jämföras med uppgifter i din patientjournal. Detta utförs av en s.k. monitor som är en speciellt tillsatt, oberoende person med tystnadsplikt. Data kan även komma att granskas av Läkemedelsverket.

Ansvarig för dina personuppgifter är Karolinska Universitetssjukhuset. Du har rätt att kontakta oss för att få information om vilka uppgifter som behandlas om dig eller för att begära rättelse, överföring, radering eller begränsning av dina personuppgifter. Du kan även kontakta vårt dataskyddsombud på Karolinska Universitetssjukhuset (dataskyddsombud.karolinska@sll.se). Du har rätt att framföra klagomål till tillsynsmyndigheten Datainspektionen om du tycker att vi behandlar dina personuppgifter på ett felaktigt sätt.

8. Hur får jag information om studiens resultat?

Du är välkommen att höra av dig till vår huvudprövare (se kontaktuppgifter nedan) för att få information om studiens resultat.

9. Försäkring och ersättning

For peer review only

Alla deltagare i studien täcks av den ordinarie patientskadeförsäkringen. Det utgår ingen separat ersättning för att delta i studien.

10. Frivillighet

Deltagande i studien är helt frivilligt och du kan när som helst, utan särskild förklaring, avbryta. Om du avbryter kommer det inte att påverka din sedvanliga vård eller behandling.

11. Ansvariga

Forskningshuvudman: Karolinska Universitetssjukhuset
Personuppgiftsansvarig: Dataskyddsombudet på Karolinska Sjukhuset

Ansvarig forskare: Professor Joakim Dillner 0724682460 Joakim.Dillner@sll.se
Klinisk Huvudprövare: Docent Johan Ursing 08-12356760 johan.ursing@sll.se
.

For peer review only

Samtyckesformulär

Avseende studien " Konvalescensplasma för behandling av COVID-19, en randomiserad kontrollerad studie"

- Jag bekräfta att jag har informerats både muntligen och skriftligen om denna studie, fått tillfälle att ställa frågor och fåt dem besvarade.
- Jag ger mitt samtycke till att delta i studien.
- Jag samtycker till att personuppgifter hanteras i överensstämmelse med den information jag fått enligt ovan inklusive att data kommer delas med studiemonitorn som får ta del av journaluppgifter för kontroll av data.
- Deltagande är helt frivilligt och kan när som helst, utan förklaring, avbrytas utan att detta påverkar den fortsatta sedvanliga vården.

For peer review only

Forskningspersonens namnteckning | Ort och datum

Forskningspersonens personnummer | Obs! Måste fyllas i av forskningspersonen!

Undertecknad har gått igenom och förklarat studiens syfte för ovanstående forskningsperson samt erhållit forskningspersonens samtycke. Forskningspersonen har även fått en kopia av forskningspersonsinformationen.

Prövarens namnteckning | Ort och datum

Ansökan om etikprövning – *Bilaga 2*
Beskrivning av biologiskt material

14. Beskrivning av biologiskt material

14.1. Kommer biologiskt material från människor att nyinsamlas för projektet?

Frågan avser ny insamling av biologiskt material (prover) från människor, dvs. material som samlas in specifikt för detta forskningsprojekt.

Ja

14.1.1. [Om ja 14.1] **Vilken eller vilka typer av biologiskt material ska samlas in?**

Ange vilken eller vilka typer av biologiskt material som ska samlas in för detta forskningsprojekt. Om alternativet Annat biologiskt material väljs ska dessa anges.

☐ Vävnad
☐ Blod
☒ Annat biologiskt material, ange vad

Serum

14.1.2. [Om ja 14.1] **Kommer det biologiska materialets ursprung gå att härleda till en enskild person, det vill säga går det att spåra eller koppla samman det biologiska materialet med den individ som materialet kommer ifrån?**

Det går att koppla samman det biologiska materialet med individen på två sätt: Antingen direkt (proverna har då märkts med identifierande uppgifter som initialer, födelsenummer eller liknande) eller genom en kodnyckel (materialet är då pseudonymiserat). Om det inte går att koppla samman det biologiska materialet med individen är materialet avidentifierat.

Ja

14.1.2.1. [Om ja 14.1.2] **Ange hur det biologiska materialet kommer att kodas.**

När ett material kodas ersätts individens identitet på materialet med en kod, och en kodnyckel tas fram. Genom kodnyckeln går det att identifiera vilken individ materialet kommer ifrån.

Proverna ges ID-nummer. ID-numret läggs in i databas som upprättas vid Karolinska Universitetslaboratoriet, där behörig personal har möjlighet att knyta det till forskningspersonens identitet.

14.1.3. [Om ja 14.1] **Hur mycket biologiskt material är planerat att samlas in?**

Ange antalet prover, antalet bitar och/eller volym som ska samlas in vid varje enskilt provtagningstillfälle. Ange även det totala antalet prover, antalet bitar och/eller den totala volymen för studien. Ange i förekommande fall även antal prover, bitar och/eller volym för varje delstudie.

Blodprov och Serumprov om 5 milliliter tas dagligen under behandlingperioden som varar 10 dagar.

14.1.4. [Om ja 14.1] **Hur länge ska det biologiska materialet vara tillgängligt för projektet?**

Ange under vilken tid forskningsprojektet kommer ha tillgång till det biologiska materialet.

2020-11-16 – 2030-11-15

14.1.5. [Om ja 14.1] **Kommer det biologiska materialet analyseras och förstöras inom sex månader efter provtagningstillfället, det vill säga är undantagsregeln tillämplig?**

Den 1 januari 2019 infördes en ny undantagsregel gällande prov som tas för forskning men som inte ska sparas i biobank. Undantagsregeln innebär att biobankslagen inte är tillämplig på prov som är avsedda för forskning och som analyseras inom sex månader efter provtagningstillfället och förstörs omedelbart efter analysen. Båda villkoren måste uppfyllas.

Även om undantagsregeln blir tillämplig i aktuellt forskningsprojekt ska alla frågor om biologiskt material besvaras med undantag för frågorna om namn och huvudman för biobanken.

Nej

For peer review only - http://bmjopen.bmj.com/site/about/guidelines.xhtml

14.1.6. [Om ja 14.1] **Ange en motivering till valet av tid som projektet kommer ha tillgång till det biologiska materialet.**

Erfarenheter av tidigare epidemier är att de ofta kommer i flera vågor, ibland under flera år. Det kan därför vara viktigt att kunna ta fram tidigare prov för parallell analys med nytagna prover. Analysmetoderna förändras snabbt och redan om något år är det troligen så stora skillnader att resultat från tidigare analyser inte kan jämföras med resultat från nya analyser på ett tillräckligt pålitligt sätt. Slutligen avser vi att publicera studien och källmaterial från publicerade studier bör vara bevarade i 10 års tid som en säkerhet för att förebygga forskningsfusk.

14.1.7. [Om ja 14.1] **Hur länge ska det biologiska materialet vara tillgängligt efter att projektet är avslutat?**

Ange under vilken tid sökanden planerar att ha tillgång till det biologiska materialet.

2020-11-16 – 2030-11-15

14.1.8. [Om ja 14.1] **Ange en motivering till valet av tid som projektet kommer ha tillgång till det biologiska materialet efter att projektet avslutats.**

Serumprov sparas för eventuell omanalys, dock längst till och med 2030-11-15

14.1.9. [Om ja 14.1] **Ange namnet på den biobank som ska ansvara för provsamlingen.**

Här avses den biobank som kommer att ansvara för den provsamling som bildas under studien, dvs. den samling som det biologiska materialet hämtas till. Ange om möjligt IVO:s registreringsnummer.

Biobank: *En samling biologiskt material från människor, förvarat för ett eller flera ändamål, samt information om detta material. En biobank kan innehålla flera provsamlingar.*

Stockholms Medicinska Biobank

14.1.10. [Om ja 14.1] **Ange huvudman för biobanken**

Ange huvudman för den biobank som ska ansvara för provsamlingen.

Huvudman: den myndighet eller organisation som juridiskt och ekonomiskt har ansvaret för viss verksamhet.

Region Stockholm

14.1.11. [Om ja 14.1] **Vilka analyser kommer att utföras på det biologiska materialet?**

Redogör för alla analyser och bearbetningar som det biologiska materialet ska genomgå.

Kvantitativ PCR och antikroppar mot Corona samt följande kemiska analyser: C-reactive protein (CRP), Procalcitonin, antal vita blodkroppar (WBC), hemoglobin (Hb), Kreatinin, Kreatine Kinas, D-dimer, Troponin, ALT, AST, LD och Bilirubin. Blodprovet efter de kemiska analyserna förstörs, medan serumprovet sparas.

14.1.12. [Om ja 14.1] **Var kommer analyserna att utföras?**

Ange var analyserna på det biologiska materialet kommer att ske. Om analyser ska ske utanför Sverige ska land/länder anges.

Olika regler gäller om prover skickas för analys inom Sverige, inom EU/ESS eller utanför EU/ESS till så kallat tredje land.

☒ I den egna verksamheten
☐ Inom Sverige
☐ Utanför Sverige, ange land/länder

Max 200 tecken

14.1.13. [Om ja 14.1] **Hur kommer det biologiska material att hanteras när analyserna är genomförda?**

Prover i en biobank som har lämnats för analys inom eller utom landet ska lämnas tillbaka till biobank i Sverige eller förstöras (kasseras) när de inte längre behövs för ändamålet. I normalfallet bör det ske inom några få år.

☒ Materialet kommer att förstöras.
☐ Materialet kommer att skickas tillbaka till biobank i Sverige.

For peer review only - http://bmjopen.bmj.com/site/about/guidelines.xhtml

14.2. Planerar projektet att använda biologiskt material från människor från en eller flera befintliga provsamlingar?

Frågan avser om man för detta forskningsprojekt kommer att begära tillgång till biologiskt material eller prover som har samlats in tidigare.

Nej

14.2.1. [Om ja 14.2] **Vilket biologiskt material är planerat att användas?**

Ange vilken typ av biologiskt material som ska användas i detta forskningsprojekt. Om alternativen "annat biologiskt material" väljs ska dessa anges.

☐ Vävnad
☐ Blod
☐ Annat biologiskt material, ange vad

[Om annat] – Max 100 tecken

14.2.2. [Om ja 14.2] **Hur mycket biologiskt material är planerat att användas?**

Ange antalet prover, bitar och/eller volymen för allt biologiskt material som är planerat att hämtas in och användas för detta projekt.

Max 2000 tecken

14.2.3. [Om ja 14.2] **Ange namnet/namnen på ansvariga biobanker och huvudmän som det biologiska materialet kommer att hämtas ifrån.**

Samtliga biobanker och huvudmän som det befintliga biologiska materialet kommer att hämtas eller begäras ut ifrån ska namnges.

Biobank: En samling biologiskt material från människor, förvarat för ett eller flera ändamål samt information om detta material. En biobank kan innehålla flera provsamlingar.

Max 2000 tecken

14.2.4. [Om ja 14.2] **Ange namnet på den biobank som kommer att ansvara för denna provsamling.**

Här avses den biobank som kommer att ansvara för den nya provsamling som bildas, dvs. den samling som det biologiska materialet hämtas till. Ange om möjligt IVO:s registreringsnummer.

Biobank: En samling biologiskt material på människor, förvarat för ett eller flera ändamål samt information om detta material. En biobank kan innehålla flera provsamlingar.

Max 200 tecken

14.2.5. [Om ja 14.2] **Ange huvudman för biobanken.**

Ange huvudman för den biobank som ska ansvara för den nya provsamlingen som bildas.

Huvudman: Den myndighet eller organisation som juridiskt och ekonomiskt har ansvaret för viss verksamhet.

Max 200 tecken

14.2.6. [Om ja 14.2] **Ange hur materialet är kodat.**

När ett material kodas ersätts individens identitet på materialet med en kod, och en kodnyckel tas fram. Genom kodnyckeln går det att identifiera vilken individ materialet kommer ifrån.

Max 2000 tecken

14.2.7. [Om ja 14.2] **Vilka analyser kommer att utföras på det biologiska materialet?**

Redogör för alla analyser och bearbetningar som det biologiska materialet ska genomgå.

Max 2000 tecken

14.2.8. [Om ja 14.2] **Var kommer analyserna att utföras?**

Ange var analyserna på det biologiska materialet kommer att ske. Om analyser ska ske utanför Sverige ska land/länder anges.

Olika regler gäller om prover skickas för analys inom Sverige, inom EU/ESS eller utanför EU/ESS till så kallat tredje land.

☐ I den egna verksamheten
☐ Inom Sverige
☐ Utanför Sverige, ange land/länder

For peer review only - http://bmjopen.bmj.com/site/about/guidelines.xhtml

[Ange land/länder] – Max 500 tecken

14.2.9. [Om ja 14.2] **Hur kommer det biologiska materialet att hanteras när analyserna är genomförda?**

Prover i en biobank som har lämnats för analys inom eller utom landet ska lämnas tillbaka till biobank i Sverige eller förstöras (kasseras) när de inte längre behövs för ändamålet. I normalfallet bör det ske inom några få år.

☐ Materialet kommer att förstöras.
☐ Materialet kommer att skickas tillbaka till biobank i Sverige.

14.2.10. [Om ja 14.2] **Hur länge ska det biologiska materialet vara tillgängligt för projektet?**

Ange under vilken tid det biologiska materialet kommer att sparas för det här forskningsprojektet.

Från och med dag månad år – Till och med dag månad år

14.2.11. [Om ja 14.2] **Ange en motivering till valet av tid som det biologiska materialet kommer vara tillgängligt för projektet.**

Max 2000 tecken

14.2.12. [Om ja 14.2] **Hur länge ska det biologiska materialet vara tillgänglig efter att projektet är avslutat?**

Ange under vilken tid sökanden planerar att ha tillgång till det biologiska materialet.

Från och med dag månad år – Till och med dag månad år

14.2.13. [Om ja 14.2] **Ange en motivering till valet av tid som det biologiska materialet ska vara tillgänglig efter att projektet är avslutat.**

Max 2000 tecken

For peer review only

For peer review only - http://bmjopen.bmj.com/site/about/guidelines.xhtml
